# Prdm6 drives ductus arteriosus closure by promoting ductus arteriosus smooth muscle cell identity and contractility

**DOI:** 10.1172/jci.insight.163454

**Published:** 2023-03-08

**Authors:** Meng Zou, Kevin D. Mangum, Justin C. Magin, Heidi H. Cao, Michael T. Yarboro, Elaine L. Shelton, Joan M. Taylor, Jeff Reese, Terrence S. Furey, Christopher P. Mack

**Affiliations:** 1 Department of Pathology and McAllister Heart Institute, University of North Carolina at Chapel Hill, Chapel Hill, North Carolina, USA.; 2 Department of Pediatrics, Vanderbilt University Medical Center, Nashville, Tennessee, USA.

**Keywords:** Vascular Biology, Cardiovascular disease, Mouse models

## Abstract

Based upon our demonstration that the smooth muscle cell–selective (SMC-selective) putative methyltransferase, Prdm6, interacts with myocardin-related transcription factor-A, we examined Prdm6’s role in SMCs in vivo using cell type–specific knockout mouse models. Although SMC-specific depletion of Prdm6 in adult mice was well tolerated, Prdm6 depletion in Wnt1-expressing cells during development resulted in perinatal lethality and a completely penetrant patent ductus arteriosus (DA) phenotype. Lineage tracing experiments in Wnt1^Cre2^ Prdm6^fl/fl^ ROSA26^LacZ^ mice revealed normal neural crest–derived SMC investment of the outflow tract. In contrast, myography measurements on DA segments isolated from E18.5 embryos indicated that Prdm6 depletion significantly reduced DA tone and contractility. RNA-Seq analyses on DA and ascending aorta samples at E18.5 identified a DA-enriched gene program that included many SMC-selective contractile associated proteins that was downregulated by Prdm6 depletion. Chromatin immunoprecipitation–sequencing experiments in outflow tract SMCs demonstrated that 50% of the genes Prdm6 depletion altered contained Prdm6 binding sites. Finally, using several genome-wide data sets, we identified an SMC-selective enhancer within the Prdm6 third intron that exhibited allele-specific activity, providing evidence that rs17149944 may be the causal SNP for a cardiovascular disease GWAS locus identified within the human PRDM6 gene.

## Graphical abstract

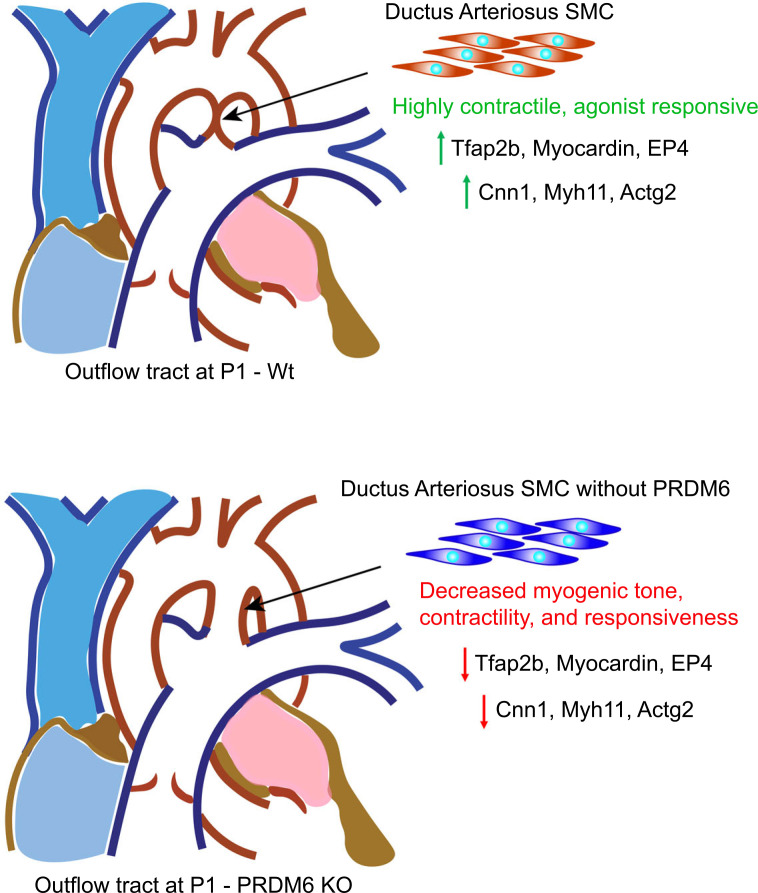

## Introduction

It is well established that the regulation of smooth muscle cell (SMC) phenotype plays an important role in cardiovascular development and disease and that serum response factor (SRF) and the myocardin family of SRF cofactors (myocardin, myocardin-related transcription factor-A, and MRTF-B) are required for the expression of most SMC differentiation marker genes. However, the absence of SMC marker gene expression in the many cell types that express high myocardin and/or MRTF levels strongly suggests that additional mechanisms are critical for the overall pattern of SMC-specific gene expression observed in vivo. One mechanism that contributes to SMC-specific gene expression is the facilitation of MRTF nuclear localization by RhoA-dependent actin polymerization. Based upon seminal studies from the Treisman lab (1, 2), we and others have shown that high levels of RhoA/MRTF signaling promote and/or maintain SMC differentiation and that this pathway serves as a critical, and perhaps integrating, mechanism by which environmental cues control SMC phenotype (3–10).

SMC-specific gene expression is also regulated by epigenetic mechanisms that alter chromatin structure and transcription factor access to DNA (11). The myocardin factors have been shown to directly recruit the histone acetyltransferase, p300 (12); the histone demethylase, Kmd3a (13); and components of the ATP-dependent SWI/SNF chromatin remodeling complex (14, 15) to the SMC-specific gene promoters. Our recent genome-wide studies on chromatin structure and transcription factor binding in human aortic SMCs have provided novel insight into the combinatorial transcription factor interactions that regulate SMC-specific gene expression and led to the identification and characterization of previously unrecognized SMC-selective proteins and regulatory elements (16–19). Nevertheless, major challenges remain in regard to the cell type and gene specificity of epigenetic regulatory mechanisms and the contributions of specific chromatin-modifying enzymes to cardiovascular development and disease.

As described more fully below, the current studies were based upon our demonstration that MRTF-A interacted with the putative methyltransferase, Prdm6. PRDI-BFI/RIZ domain proteins are defined by the presence of a PR domain that shares high homology to the methyltransferase SET domain of the Suvar/Enhancer of zest/Trithorax family and the presence of a variable number of Zn fingers thought to mediate protein-protein and protein-DNA interactions. PRDM proteins have been shown to regulate cell fate decisions by functioning as histone methyltransferases and/or by interacting with positive and negative chromatin-remodeling enzymes (see ref. 20 for a review). Of importance, Prdm6 expression is highly SMC selective in mice (21), and its deletion in SM22-expressing cells during development results in perinatal lethality and pulmonary hemorrhage (22). Further supporting the importance of Prdm6 in SMCs in human cardiovascular disease, a genetic study described Prdm6-coding mutations that were associated with patent ductus arteriosus (DA) (23). Finally, a number of GWAS have identified noncoding variants within the Prdm6 gene that are associated with multiple cardiovascular disease endpoints, including blood pressure and intracranial aneurysm (24–29). The causal variant(s) that mediates these effects has not been identified.

The goals of the current study were to better assess Prdm6’s role in SMCs in vivo, to identify the mechanisms by which Prdm6 affects SMC function, and to characterize the transcriptional mechanisms and genetic variants that regulate its SMC-selective expression.

## Results

### Prdm6 interacts with MRTF-A.

**Figure 1:**
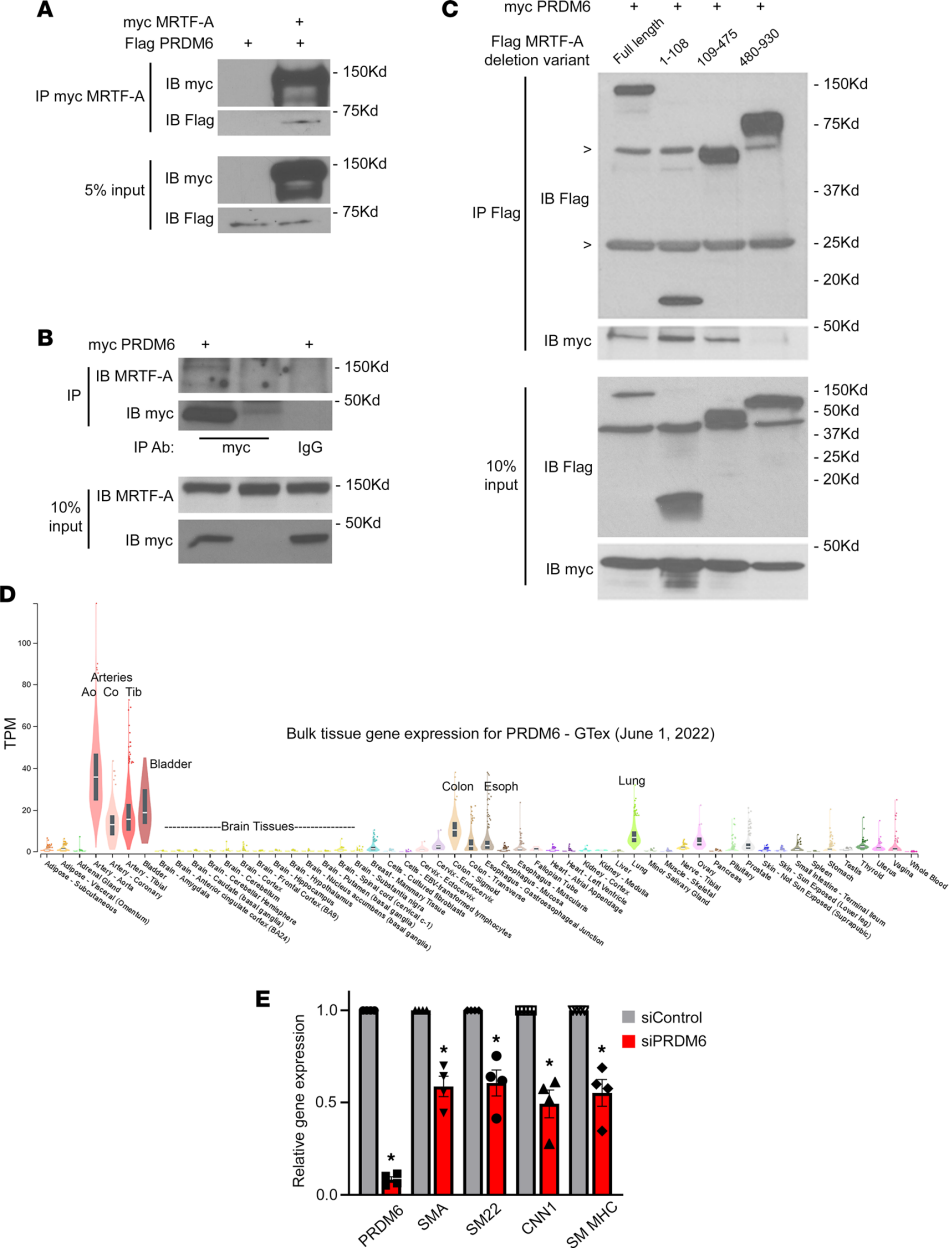
Prdm6 is an SMC-selective MRTF-A binding protein that regulates SMC phenotype. (**A**) COS-7 cells were transfected with flag-Prdm6 with/without myc-MRTF-A. Myc immunoprecipitates were separated on an SDS-PAGE gel, transferred to nitrocellulose, and then probed with anti-flag Ab. *n* = 2; representative blots shown. (**B**) 10T1/2 cells were transfected with myc-Prdm6 or empty vector. Myc and control IgG immunoprecipitates were run on SDS-PAGE and probed with an Ab against endogenous MRTF-A. Note that MRTF-A was only detected in IPs from lysates expressing myc-Prdm6 and immunoprecipitated with the anti-myc Ab. *n* = 2; representative blots shown. (**C**) COS-7 cells were transfected with myc-Prdm6 and the indicated flag-MRTF-A deletion fragment. Flag immunoprecipitates were run on an SDS-PAGE gel and probed with anti-flag (IP) or anti-myc (Co-IP) Abs. Nonspecific bands for IgG heavy and light chains are marked with arrowheads. *n* = 2; representative blots shown. (**D**) Genotype-Tissue Expression (GTEx) consortium data depicting normalized Prdm6 mRNA levels in the indicated human tissues. (**E**) Rat AoSMCs were treated with siRNAs targeting Prdm6 or GFP for 72 hours. Expression of the indicated genes was analyzed by quantitative reverse transcription PCR and normalized to GAPDH. *n* = 4; *P < 0.05. TPM, transcripts per million; SMA, SM α-actin.

In an attempt to uncover novel mechanisms that regulate SMC-specific gene expression, we used co-immunoprecipitation/mass spectrometry–based methods to identify MRTF-A binding partners in mouse aortic SMC (AoSMC) lysates. The SMC-selective PR/SET domain-containing protein, Prdm6, was identified in washed MRTF-A immunoprecipitates, as were several known MRTF-A interacting proteins including p300 (12, 30) and importin 9, which facilitates MRTF-A entry into the nucleus (31). (See Supplemental Table 1; supplemental material available online with this article; https://doi.org/10.1172/jci.insight.163454DS1.) Although the lack of a suitable PRDM6 Ab prevented us from examining the interaction between endogenous proteins, both flag-MRTF-A (Figure 1A) and endogenous MRTF-A (Figure 1B) were shown to co-immunoprecipitate with exogenously expressed myc-Prdm6. To further validate and characterize the MRTF-A–Prdm6 interaction, we performed co-immunoprecipitation experiments in COS-7 cells overexpressing a series of MRTF-A deletions. As shown in Figure 1C, myc-Prdm6 interacted with the central region of MRTF-A (aa 109–475) that contains the B1 basic, Q-rich, and SAP domains known to mediate SRF binding, and with an N-terminal fragment of MRTF-A (aa 1–108) that contains the actin-binding REPEL motifs. The MRTF-A transaction domain (aa 480–930) did not interact with Prdm6. Since the formation of large actin-containing binding complexes in cell lysates frequently complicates co-immunoprecipitation results, we used far Western analyses to further test whether the MRTF-A–Prdm6 interaction was direct. As shown in Supplemental Figure 1A, we detected direct binding of Prdm6 only to full-length MRTF-A and the aa 109–475 region (see arrows). In separate experiments, the association of Prdm6 with the N-terminal region of MRTF-A was inhibited by inclusion of cytochalasin D, suggesting that this interaction was actin dependent, and likely nonspecific (Supplemental Figure 1B).

Prdm6 was originally characterized by Davis et al. as a protein enriched in mouse SMCs (21). Interestingly, *PRDM6* mRNA levels in humans were highest in blood vessels and other SMC-containing organs (see data from the Genotype-Tissue Expression [GTEx] consortium presented in Figure 1D), providing additional support for PRDM6’s role in SMCs. Although PRDM6 was shown to promote SMC phenotypic modulation, perhaps by interacting with HDACs and/or G9a (21), Gewies et al. detected no significant differences in SMC proliferation, SMC marker gene expression, or SMC investment of vessels in SM22^Cre^ Prdm6^fl/fl^ mouse embryos (22), suggesting that the effects of Prdm6 on SMC phenotype were context dependent. Indeed, PRDM proteins have been shown to interact with multiple chromatin-modifying enzymes and to mediate both transcriptional activation and repression depending upon cellular and tissue context (see ref. 32 for a review). Given that Prdm6 interacted with MRTF-A, a strong transactivator of SMC marker gene expression, we wanted to test whether Prdm6 depletion affected SMC differentiation marker gene expression in our cultured SMC models. As shown in Figure 1E, siRNA-mediated depletion of Prdm6 by approximately 90% in primary rat AoSMCs resulted in a 40% reduction in mRNA levels of several canonical SMC markers (*P* < 0.05). SMC differentiation marker protein levels were also reduced when primary mouse outflow tract SMCs isolated from Prdm6^fl/fl^ animals were treated with Cre-expressing adenovirus (Supplemental Figure 2).

### Prdm6 expression in neural crest–derived SMCs is required for DA closure.

**Figure 2:**
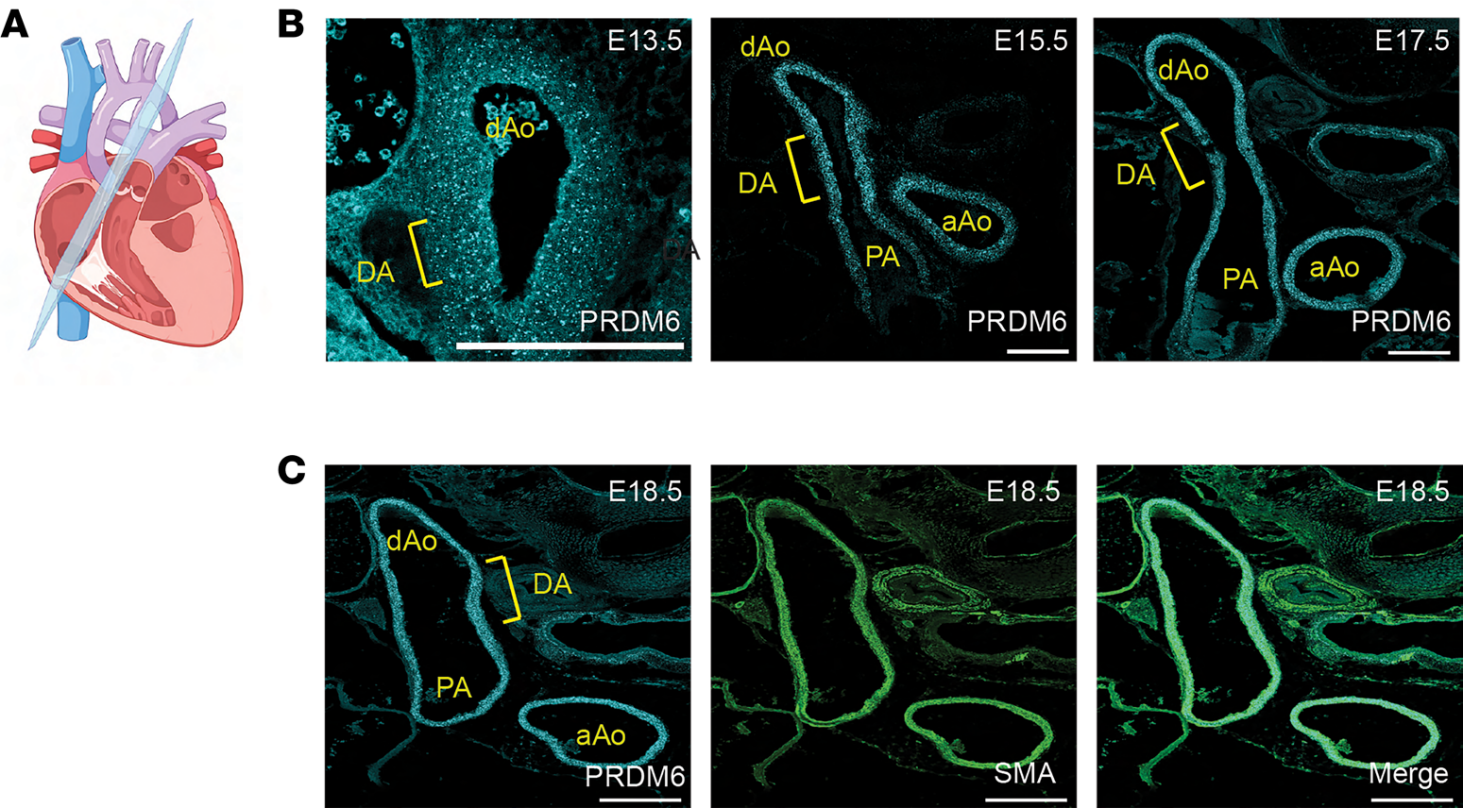
Prdm6 is highly expressed in outflow tract SMCs during development. (**A**) Schematic of section orientation for DA and outflow tract analysis. (**B**) RNAscope-based in situ hybridization for Prdm6 shows expression from E13.5 to E17.5 in all outflow tract arteries including the ascending aorta (aAo), descending aorta (dAo), pulmonary artery (PA), and DA. At least 2 pups per time point were examined. Representative images are shown. (**C**) Costaining of Prdm6 and SM α-actin (SMA) at E18.5 verified that Prdm6 expression is specific to vascular SMCs. Scale bar = 200 μm.

Because Prdm6 was shown to be expressed in outflow tract SMCs (21) and because coding mutations in Prdm6 were associated with patent DA in humans (23), we wanted to directly examine the role of Prdm6 in outflow tract development and DA function. We first used RNAscope-based methods to more closely monitor Prdm6 expression during outflow tract maturation starting just after separation of the aortic and pulmonary arteries at E13.5. As shown in Figure 2 and Supplemental Figure 3, using RNAscope probes to PRDM6 resulted in a strong and specific in situ signal in the SMCs of the aortic arch, pulmonary artery, and DA, from E13.5 until just before birth.

**Figure 3:**
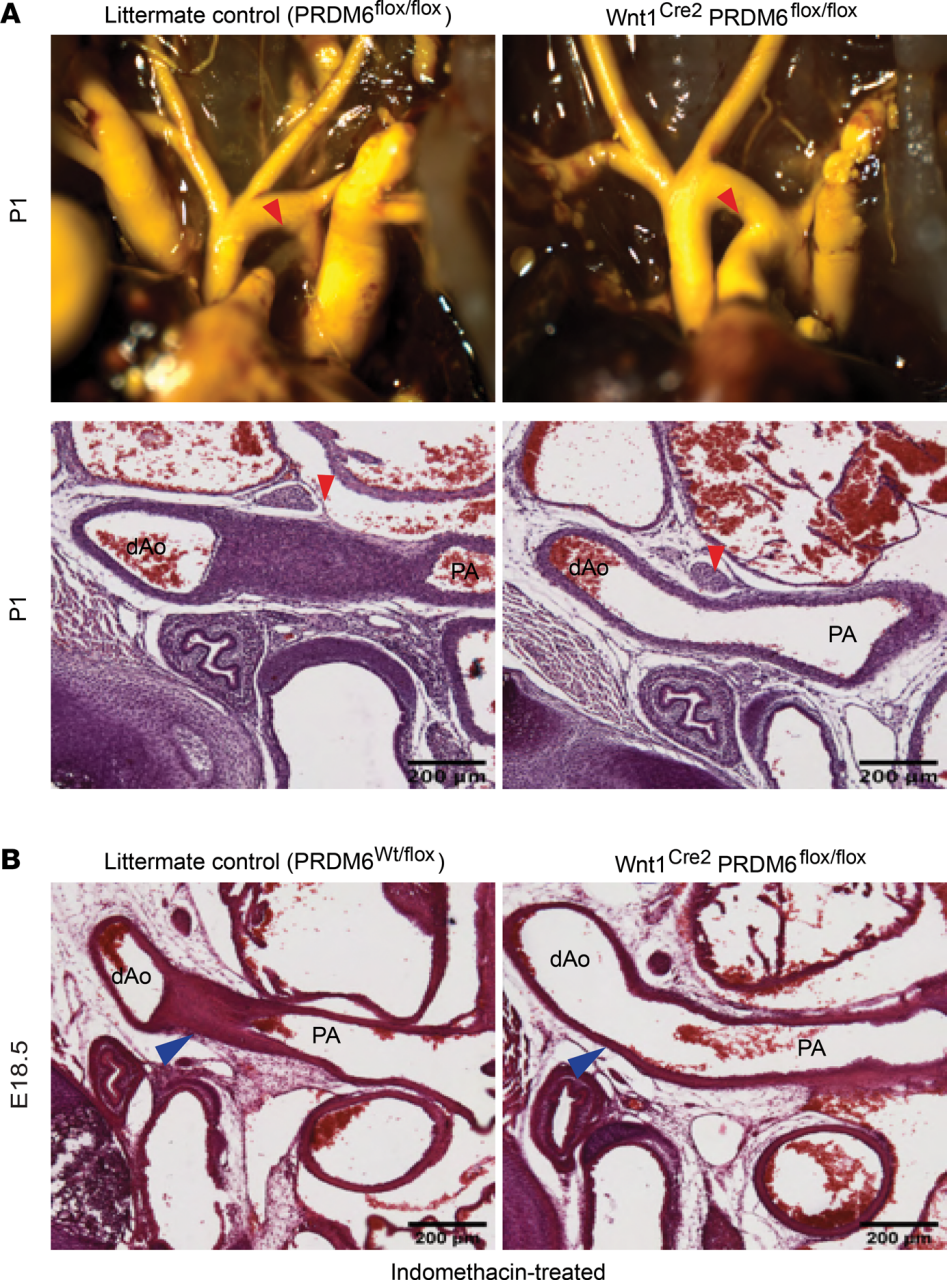
Wnt1^Cre2^ Prdm6^fl/fl^ mice exhibit patent DA. (**A**) Intracardiac Microfil injection (top) and histologic examination (bottom) of DA closure in Wnt1^Cre2^ Prdm6^fl/fl^ and littermate control mice at P1. The DA in these images is marked by a red arrowhead. At least 6 pups per group were examined. Representative images are shown. (**B**) Histological analysis of DA closure in E18.5 embryos isolated from dams treated for 4 hours with indomethacin (20 mg/kg). As marked by blue arrowheads, note that indomethacin treatment resulted in premature DA closure in utero in littermate control but not Wnt1^Cre2^ Prdm6^fl/fl^ mice. Scale bar = 200 μm. At least 7 fetuses per group were analyzed. Representative images shown. dAo, descending aorta; PA, pulmonary artery.

To directly test whether Prdm6 was required for normal neural crest-derived SMC development and function, we bred Prdm6^fl/fl^ mice to a Wnt1^Cre2^ line that expresses Cre in neural crest cells starting at approximately E10.5 (33). Wnt1^Cre2^ Prdm6^fl/fl^ mice were born at Mendelian ratios, and as shown in Supplemental Figure 4, were indistinguishable from littermate controls, suggesting that Prdm6 depletion in neural crest cells did not result in embryonic lethality or have gross effects on overall development. In contrast, no Wnt1^Cre2^ Prdm6^fl/fl^ mice survived until weaning even though 53 of the 266 offspring generated were expected to have this genotype. Based upon our observations during the perinatal period, Wnt1^Cre2^ Prdm6^fl/fl^ mice died within 2 days of birth, a phenotype similar to that observed in the SM22^Cre^ Prdm6^fl/fl^ model (22) and one that is common in genetically modified mouse models of patent DA (34). We carefully examined DA patency in newly born Wnt1^Cre2^ Prdm6^fl/fl^ mice (before lethality) by visual scoring of blood within the DA, by outflow tract casting, and by microscopic analysis of paraffin-embedded and frozen sections. As depicted in Figure 3, we observed a patent DA in all Wnt1^Cre2^ Prdm6^fl/fl^ mice, but not in littermate control animals, which included the following genotypes: Wnt1^Cre2^ PRDM6^wt/fl^, Wnt1^Cre2^ PRDM6^wt/wt^, PRDM6^fl/fl^, PRDM6^wt/fl^, and PRDM6^wt/wt^. Taken together these data indicated that PRDM6 expression was absolutely required for DA closure, that Cre expression alone or deletion of 1 copy of PRDM6 had no effect on ductus closure, and that the floxed PRDM6 allele behaved in a manner similar to the WT allele.

Functional DA closure in mice is initiated within minutes after birth and is mediated by severe vessel constriction. Full anatomic closure takes place over a longer period and involves intimal thickening and eventual fibrotic remodeling of the DA into a remnant structure known as the ligamentum arteriosum (35). Based upon the timing of lethality in our model and the fact that we never observed even partial ductus closure in Wnt1^Cre2^ Prdm6^fl/fl^ mice, we hypothesized that Prdm6 expression in SMC was required for the contractile pathways that mediate DA vasoconstriction. To begin to test this idea, we stimulated ductus closure in E18.5 embryos by treating pregnant dams for 4 hours with indomethacin (20 mg/kg), a nonsteroidal antiinflammatory drug used to treat patent DA in humans because it inhibits the production of prostaglandin E_2_ (PGE_2_), which helps maintain ductus patency by stimulating ductus SMC relaxation. While this procedure resulted in premature DA closure in all littermate controls, it had no effect on DA closure in Wnt1^Cre2^ Prdm6^fl/fl^ fetuses (Figure 3B). We also subjected E18.5 newborns (delivered by cesarean section, C-section) to 100% oxygen, which is known to stimulate ductus SMC contraction by mechanisms not yet fully described (see refs. 36, 37 for reviews). As with indomethacin treatment, oxygen exposure for 1 hour did not stimulate DA closure in Wnt1^Cre2^ Prdm6^fl/fl^ animals (data not shown). Importantly, we observed functional DA closure of WT mice within 30 minutes of the C-section procedure even under ambient air conditions.

### Neural crest cell investment of the outflow tract is unaffected by Prdm6 depletion.

**Figure 4:**
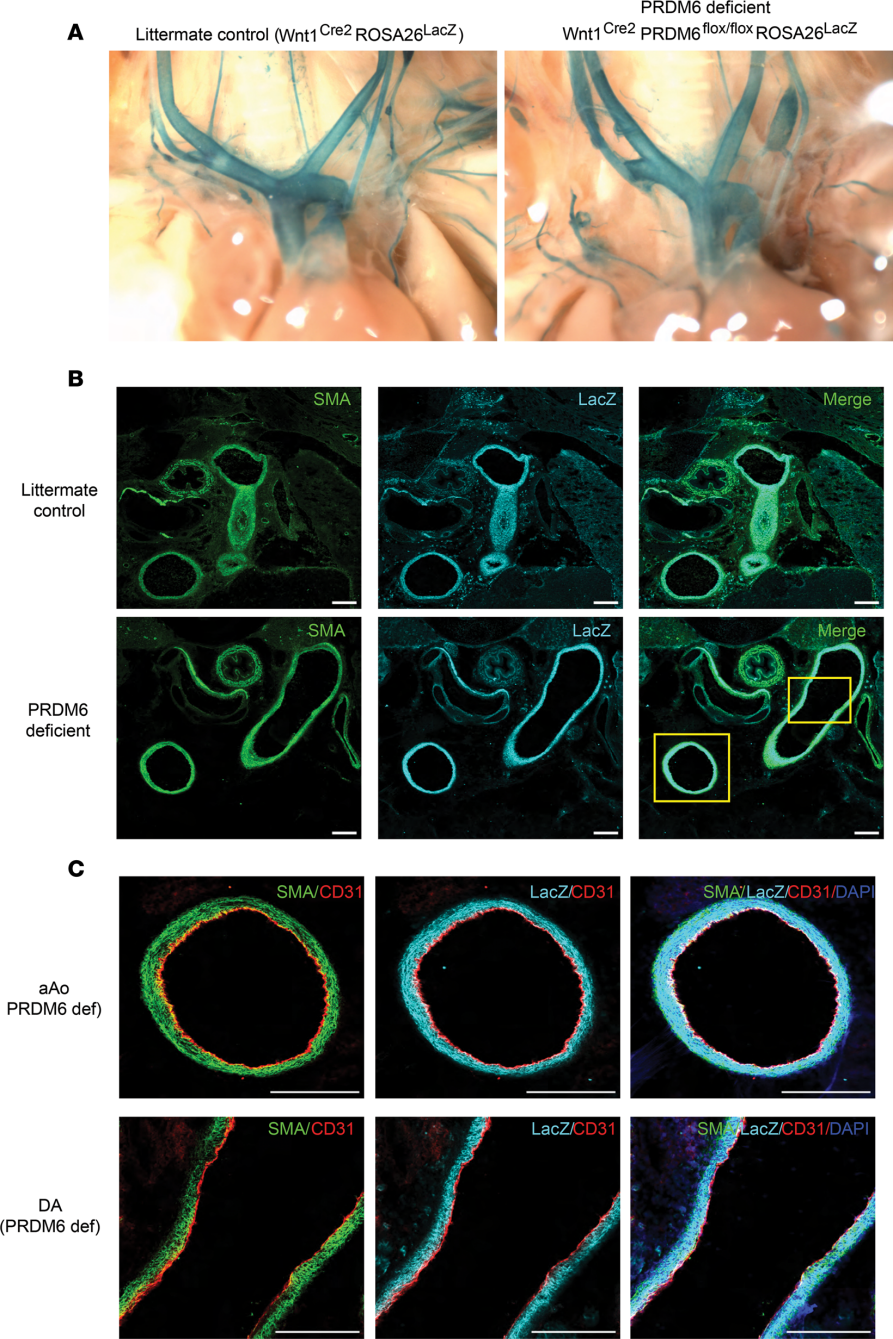
Prdm6 depletion does not affect neural crest cell investment of the outflow tract arteries. (**A**) X-gal staining of outflow tract arteries in Wnt1^Cre2^ ROSA26^LacZ^ and Wnt1^Cre2^ Prdm6^fl/fl^ ROSA26^LacZ^ pups at P1. At least 4 pups per group were examined. Representative images are shown. (**B**) Immunofluorescence staining for SM α-actin (SMA) and LacZ in P1 outflow tract sections from littermate control and neural crest–specific Prdm6-deficient mice. At least 4 pups per group were examined. Representative images are shown. Scale bar = 200 μm. (**C**) The boxed area in panel **B** is shown at higher magnification. SMA (green), LacZ (cyan), CD31 (red), and DAPI (blue). Scale bar = 200 μm. def, deficient.

In our model, the Wnt1 promoter drives Cre expression specifically in neural crest cells relatively early during development. Since cardiac neural crest cells delaminate from the developing neural tube and migrate into the pharyngeal arches, where they condense and eventually differentiate into the SMCs that layer the outflow tract and aortic arch arteries, including the DA, the defects in DA closure observed could have been due to failure of neural crest cells to correctly populate these vessels. To address this possibility, we crossed our Wnt1^Cre2^ Prdm6^fl/fl^ mice to the ROSA26^LacZ^ reporter strain, which allowed us to perform lineage tracing of WT and Prdm6-deficient neural crest cells during development. As shown in Figure 4A, X-gal staining of outflow tract and aortic arch arteries from Wnt1^Cre2^ Prdm6^fl/fl^ ROSA26^LacZ^ pups at P1 was consistent with normal neural crest cell migration and was not detectably different from littermate controls (Wnt1^Cre2^ ROSA26^LacZ^). To further examine the effects of Prdm6 depletion on outflow tract morphology and neural crest cell identity, we stained frozen sections from P1 pups with anti-LacZ and anti–SM α-actin Abs. As shown in Figure 4, B and C, LacZ and SM α-actin expression in the outflow tract and DA of Wnt1^Cre2^ Prdm6^fl/fl^ ROSA26^LacZ^ mice was similar to that of genetic littermate controls. Importantly, virtually all cells within the medial layers of the outflow tract vessels including the DA costained for LacZ and SM α-actin, and we observed no visible immunofluorescence in any sections with secondary Ab alone (see Supplemental Figure 5). Taken together these data suggest that Prdm6 depletion in Wnt1-expressing cells did not affect neural crest delamination or migration or have observable effects on the investment of the outflow tract arteries and DA with SM α-actin–expressing, neural crest cell–derived SMC.

### Prdm6 depletion inhibits DA contractility.

**Figure 5:**
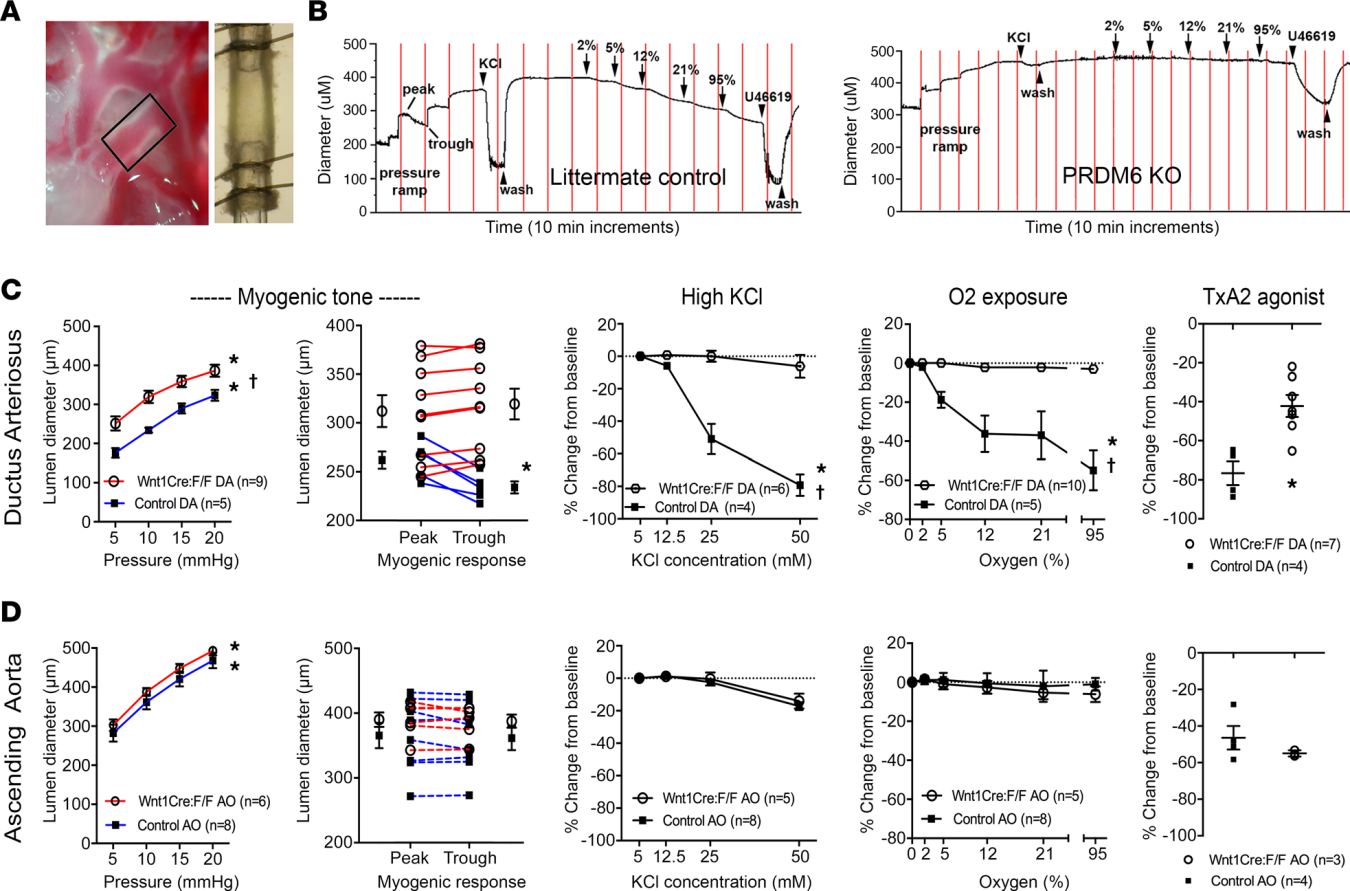
Prdm6 depletion reduces DA tone and contractile responses. (**A**) Visualization of the DA vessel segment isolated for myography experiments. (**B**) Representative diameter tracings for littermate control and Prdm6-deficient DAs exposed to increasing hydrostatic pressure, KCl, oxygen, and the thromboxane (TxA2) agonist, U46619 (100 nM). Please see Methods for more details. Summary graphs for the indicated exposures in DA (**C**) and ascending aorta (**D**) vessel segments. Note that Prdm6 deficiency did not affect the function of ascending aorta segments. *P < 0.05 for pressure and concentration response curves compared with baseline values (ANOVA) or U46619 response (*t* test); ^†^*P* < 0.05 between Wnt1^Cre2^ Prdm6^fl/fl^ and control response curves (2-way ANOVA).

To better examine the functional consequences of Prdm6 deletion on outflow tract vascular function, DA and ascending aorta segments were isolated from Wnt1^Cre2^ Prdm6^fl/fl^ and littermate controls at E18.5 and then cannulated for pressure myography as previously described (38–41). In brief, vessel segments were exposed to stepwise increases in intralumenal pressure followed by various contractile stimuli while computer-assisted video microscopy was used to record vessel diameter (see Figure 5A). When compared with littermate controls, DA segments isolated from Wnt1^Cre2^ Prdm6^fl/fl^ mice were wider in diameter under basal (5 mmHg) and working pressures (20 mmHg) and failed to exhibit a myogenic (i.e., distension-induced) contractile response (Figure 5, B and C). Ascending aorta diameter was similar between Wnt1^Cre2^ Prdm6^fl/fl^ and littermate controls under these conditions, and neither control nor Prdm6-deficient ascending aorta segments exhibited a myogenic response (Figure 5D).

To test whether Prdm6 deficiency inhibited depolarization-dependent contractility, vessel segments were exposed to increasing concentrations of KCl that are known to activate voltage-dependent calcium channels. While 50 mM KCl resulted in an 80% decrease in the diameter of DA segments isolated from littermate controls, it had virtually no effect on DA segments isolated from Wnt1^Cre2^ Prdm6^fl/fl^ mouse embryos (Figure 5C). Based upon extensive studies demonstrating that increasing oxygen concentrations promote DA constriction, we also tested whether Prdm6 deficiency affected this contractile mechanism. Time-limited exposures to increasing oxygen concentrations in the circulating buffer resulted in significant constriction of control DA segments as expected (Figure 5C), but this response was completely absent in Prdm6-depleted DA segments. Vessel segments (under oxygenated conditions) were also exposed to the thromboxane receptor agonist, U46619, which potently constricts most blood vessels through GPCR-coupled signaling pathways. Importantly, U46619 treatment of DA segments isolated from Prdm6-deficient mice resulted in a 40% reduction in vessel diameter, providing reassurance that our Prdm6-deleted vessel preparations were viable and could generate a significant contractile response under at least some conditions. However, as above, U46619-induced constriction of Prdm6-deficient DA segments was significantly less than that measured in DA segments isolated from littermate controls (–42.2% vs. –76.5%, *P* < 0.05). Of interest, Prdm6 depletion had no effect on U46619-induced contraction in ascending aorta segments or on the relatively modest constriction induced in aortic segments by KCl and oxygen exposure (Figure 5D). Taken together, these data indicate that DAs from Wnt1^Cre2^ Prdm6^fl/fl^ mice have reduced tone and innate contractility and that these properties are similar to those measured in the ascending aorta at this gestational time point (E18.5).

### Prdm6 expression is required for enrichment of a DA-selective gene program.

**Figure 6:**
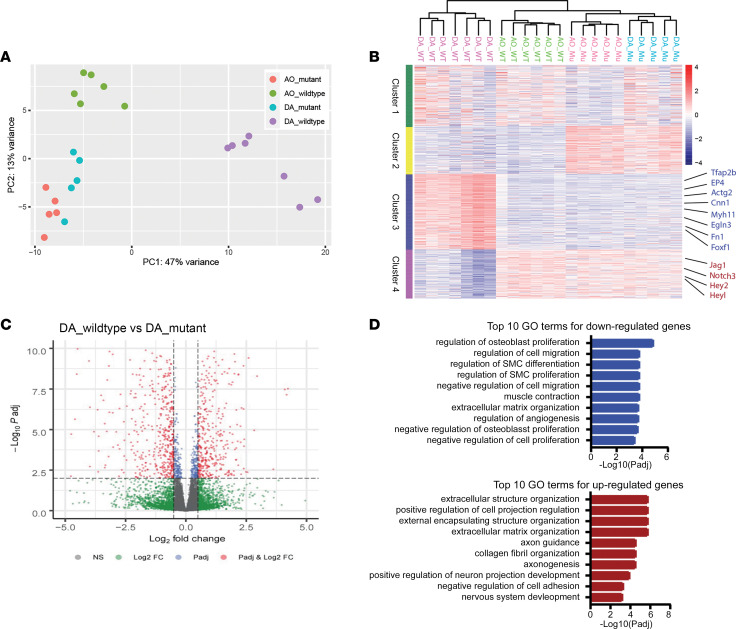
Prdm6 depletion inhibits a ductus-selective gene program. (**A**) Principal component analysis of RNA-Seq data from ascending aorta and DA samples isolated from Wnt1^Cre2^ Prdm6^fl/fl^ and littermate control mice at E18.5. (**B**) Two-dimensional hierarchical clustering of the top 1,000 genes differentially expressed in ascending aorta and DA samples from control and Prdm6-deficient mice. Potentially relevant Prdm6-dependent genes are listed on the right, and full gene lists for each cluster are available in Supplemental Table 2. (**C**) Volcano plot demonstrating differential gene expression between control and Prdm6-deficient DA samples. Genes colored in red are considered as significantly different (*P*adj < 0.01, logFC > 0.5, or logFC < –0.5). (**D**) Gene ontology analysis of genes significantly up- or downregulated by Prdm6 depletion in DA samples. logFC, log fold-change.

To help deduce the mechanisms by which Prdm6 affected DA contractility, we performed bulk RNA-Seq analysis on DA tissue isolated from Wnt1^Cre2^ Prdm6^fl/fl^ and littermate control mice at E18.5 (before DA closure). We also isolated mRNA from the ascending aorta to better define the differences between these 2 neural crest–derived SMC populations and to test whether Prdm6 had vessel-specific effects. Importantly, we sequenced at least 5 samples for each genotype and tissue, which increased the statistical power of subsequent comparisons. As summarized in the principal component analysis and hierarchical clustering in Figure 6, A and B, we saw excellent agreement in expression profiles between samples, allowing us to draw several important conclusions. First, even though DA and ascending AoSMCs originated from similar neural crest cell progenitors, we observed significant differences in gene expression patterns between these tissues. Interestingly, mRNA levels for SRF and nearly all the SRF-dependent SMC differentiation marker genes (SM MHC, SM α-actin, SM γ-actin, Cnn1, SM22, and MLCK/telokin) were significantly higher in DA samples, indicating that strong SMC identity may be critical for DA function.

Second, as illustrated by clusters 3 and 4 in Figure 6B and the volcano plot in Figure 6C, the depletion of Prdm6 in neural crest cells resulted in a significant shift in DA gene expression patterns, especially in those genes that distinguish the DA from the ascending aorta. Of the 519 genes that were more highly expressed in the DA by at least 1.5-fold, 319 of those were significantly (*P* < 0.01) downregulated by Prdm6 depletion. Similarly, of the 399 genes that exhibited lower expression in the DA (less than 70% versus aorta), 228 of those were significantly upregulated by Prdm6 depletion. Gene Ontology analysis of the most significant differentially expressed genes between control and Wnt1^Cre2^ Prdm6^fl/fl^ mice (*P*adj < 0.01 and logFC > 0.5 or logFC < –0.5) revealed that the SMC differentiation, SMC proliferation, and muscle contraction gene programs were downregulated in the Prdm6-deficient DA while those related to nerve function and extracellular matrix expression and organization were upregulated (Figure 6D). Third, Prdm6 depletion altered the expression level of many genes previously implicated in DA closure (see ref. 34 for a review), including the PGE_2_ receptor, EP4; the transcription factors Tfap2b, myocardin, Foxc1, and Hand2; the Notch signaling components, Jag1 and Notch3; SM MHC; integrin linked kinase; and fibulin1. Finally, when coupled with our demonstration that Prdm6 inhibited DA contractility, our RNA-Seq data expand the list of candidate genes that may regulate DA function to include those genes that are involved with voltage-dependent excitation contraction coupling and oxygen sensing.

**Figure 7:**
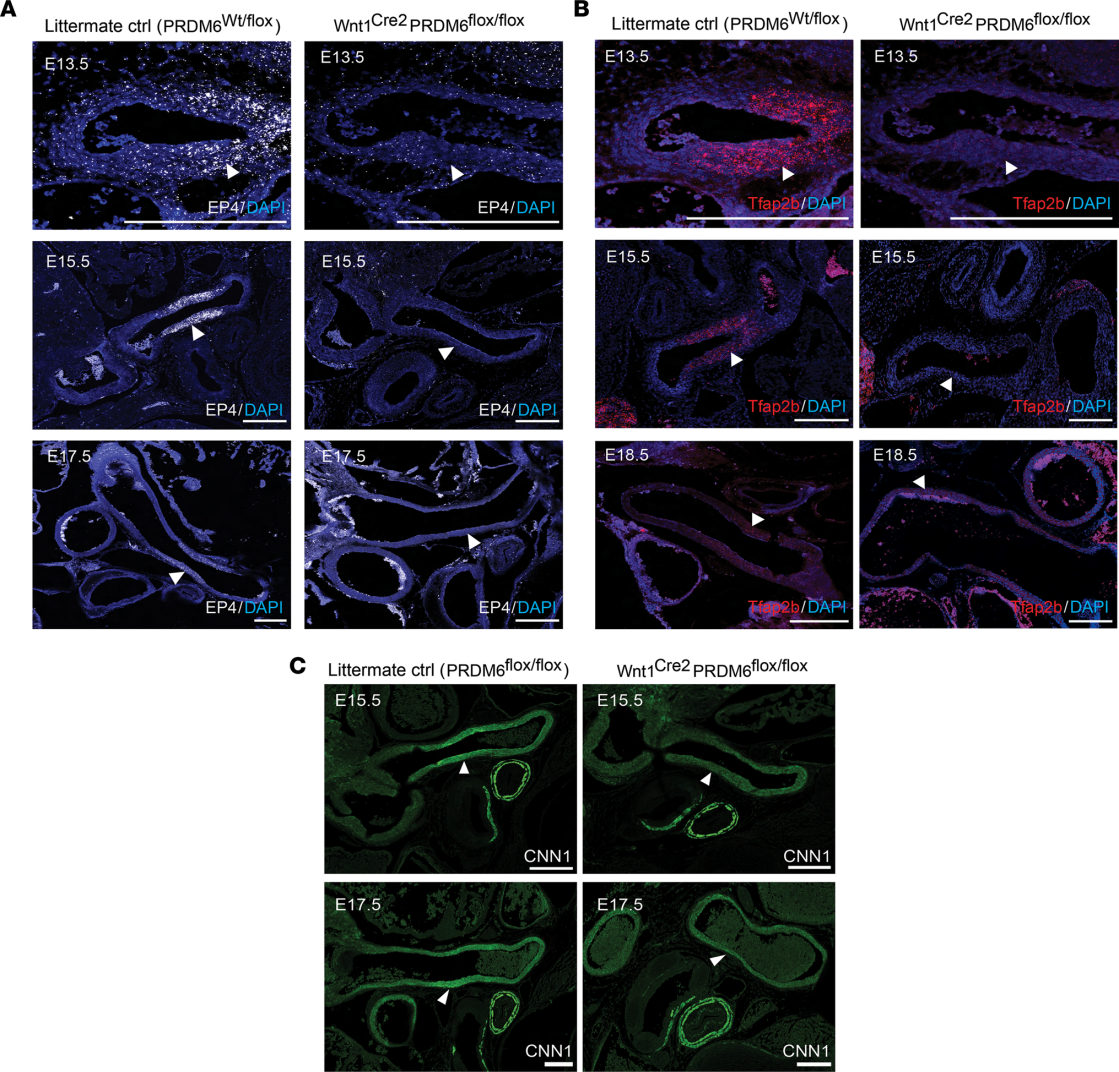
Characterization of EP4, Tfap2b, and CNN1 expression in littermate control and Prdm6-deficient neural crest–derived SMCs. RNAscope-based in situ hybridization of EP4 (**A**) and Tfap2b (**B**) expression in outflow tract arteries at the indicated developmental time points. At least 3 pups per group per time point were examined by these methods. Representative images are shown. (**C**) Immunofluorescence staining of CNN1 in outflow tract arteries at E15.5 and E17.5. At least 3 pups per group per time point were examined. Representative images are shown. Scale bar = 200 μm. For all panels note the Prdm6-dependent expression of each gene in the DA (white arrowheads).

To help support our RNA-Seq data, we used RNAscope approaches to monitor the expression of Tfapb2 and EP4 during outflow tract development in Wnt1^Cre2^ Prdm6^fl/fl^ and littermate control mice. As shown in Figure 7 and Supplemental Figure 6, our RNAscope methods resulted in specific in situ signal that was fairly selective for ductus SMCs and was mostly abolished by Prdm6 depletion. Although the increased expression of Cnn1 protein in the DA was also reduced by PRDM6 depletion (Figure 7C), we had difficulty detecting differences in SM α-actin and SM MHC protein expression by immunofluorescence. Although we cannot rule out discrepancies between mRNA and protein levels, this was most likely due to the limits of immunofluorescence quantification when examining highly expressed proteins.

### Characterization of Prdm6 DNA binding by ChIP-Seq.

**Figure 8:**
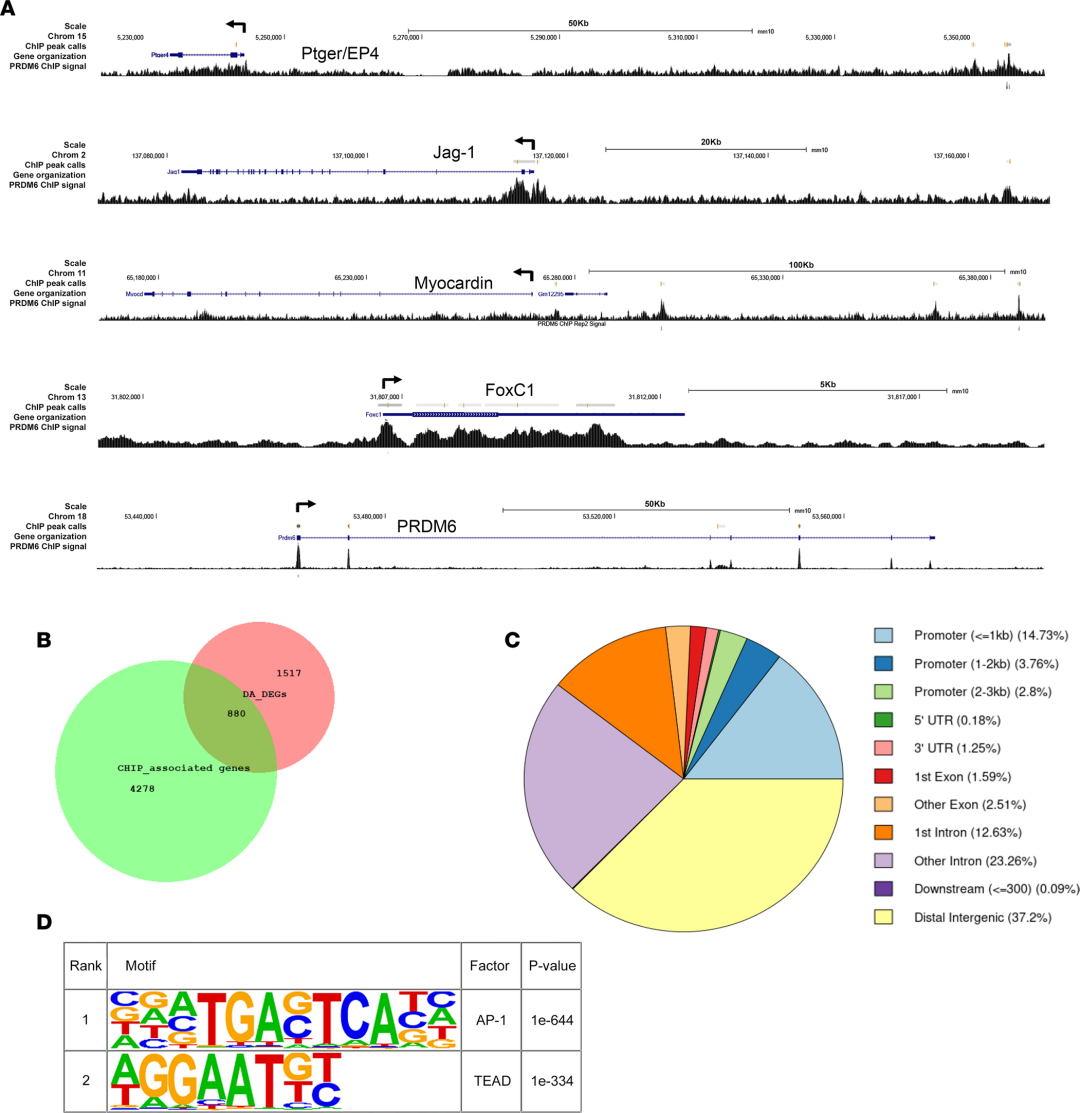
ChIP-Seq analysis of Prdm6 binding in mouse outflow tract SMCs. (**A**) Schematic of Prdm6 ChIP-Seq binding data for the indicated genes. (**B**) Venn diagram illustrating overlap of genes that bind Prdm6 (green circle) and those shown to be differentially expressed in ductus samples from littermate control and Wnt1^Cre2^ Prdm6^fl/fl^ mice (red circle). (**C**) Characterization of Prdm6 binding by gene region. (**D**) The top 2 overrepresented cis binding elements in the Prdm6 ChIP-Seq data set.

Regardless of whether Prdm6 functions as a direct histone methyltransferase, data from the current and previous studies indicate the Prdm6 has substantial effects on gene expression, suggesting that it interacts with DNA directly (perhaps through its 4 Zn fingers) or is recruited as part of a larger DNA binding complex. To further identify the mechanisms by which Prdm6 controls gene expression, we performed ChIP-Seq experiments in outflow tract SMCs isolated from E17.5 mouse embryos to identify Prdm6 binding sites within the SMC genome. Since suitable Abs for Prdm6 are not available, we used lentivirus to express flag-Prdm6 in our cells. Our final data set included only ChIP peaks detected in 2 separate immunoprecipitations from the same sample. We observed nearly 14,000 Prdm6 ChIP peaks that were associated with approximately 5,200 genes. Of the approximately 1,500 genes that were differentially expressed in Prdm6-deficient ductus tissue, about half were associated with Prdm6 binding (Figure 8B). Although these data indicate that Prdm6 affects the transcription of these genes, it also suggests that Prdm6’s effects on gene expression are modified by additional transcription and/or chromatin signals. Importantly, Prdm6 binding was detected within or near several genes known to regulate ductus function, including EP4, endothelin-1, Jag1, Connexin 40, and the transcription factors Myocd, Foxc1, and Twist1 (Figure 8A). The Prdm6 gene itself contained 4 Prdm6 binding sites, perhaps suggesting feedback regulation of its activity. Somewhat surprisingly we did not observe many Prdm6 binding sites within the SMC differentiation marker genes, though 1 strong peak was present at the SRF-dependent enhancer within the SM α-actin first intron that we have previously characterized (42).

A breakdown of Prdm6 binding by gene component (Figure 8C) demonstrated that Prdm6 was frequently associated with transcription start sites, suggesting that it plays a role in transcription initiation. However, Prdm6 binding was even more prevalent downstream of the transcription start site (TSS), especially in early introns, raising the possibility that it has additional roles in transcription maintenance or processing. Bioinformatic comparison of the Prdm6 binding regions revealed several overrepresented sequences that could reflect direct Prdm6 binding or its interaction with additional transcription factors (Figure 8D). Of interest was a TTTC/AT sequence that was identified as a potential Prdm6 binding site by Schmitges et al., who performed a large-scale ChIP-Seq screen on 78 Zn finger–containing proteins overexpressed in HEK293T cells including Prdm6 (43). Consensus sequences for AP1 and TEAD transcription factors were also detected by this comparison.

### Transcription mechanisms that control Prdm6 expression.

**Figure 9:**
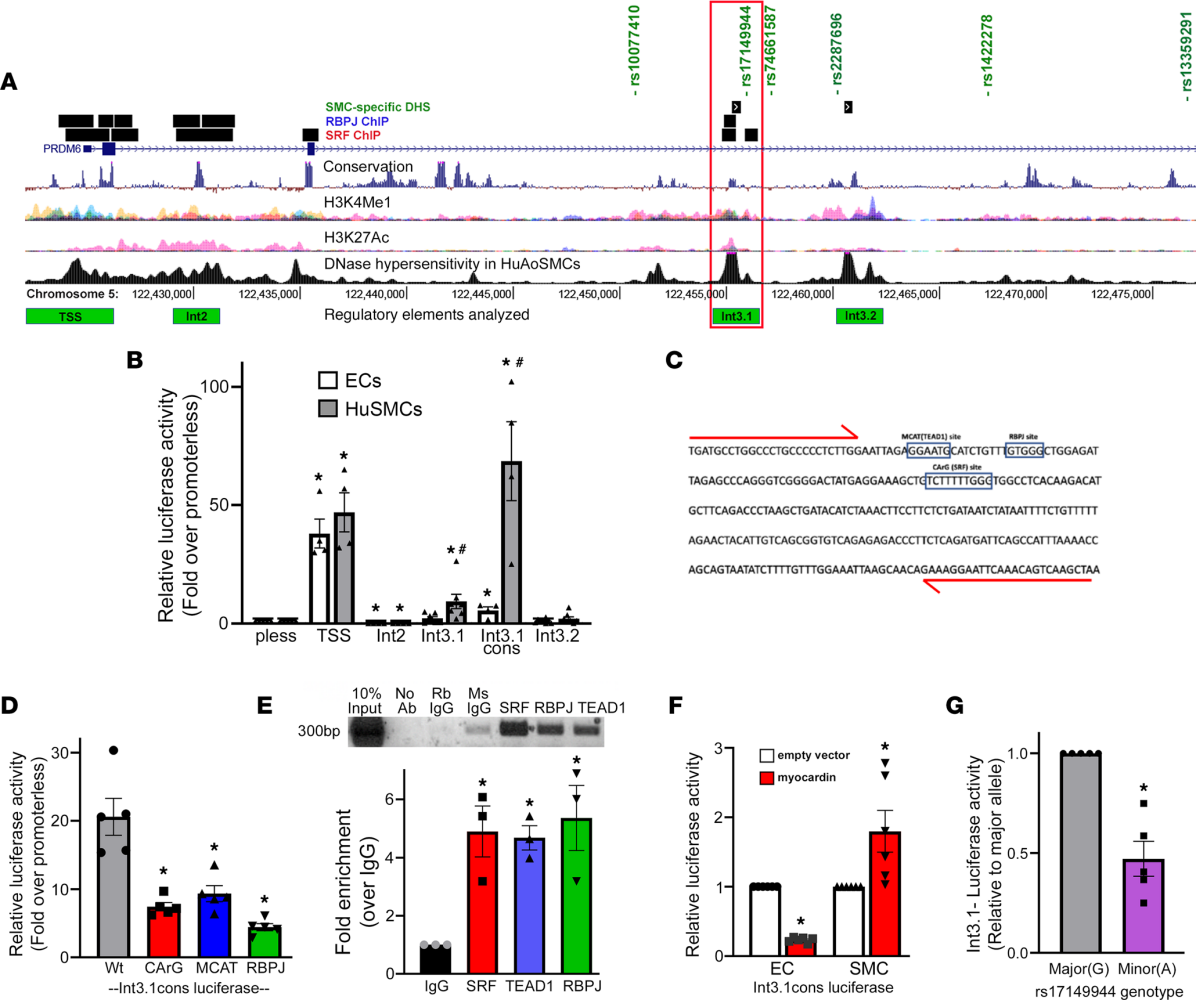
Identification of regulatory elements and genetic variations that control Prdm6 expression in SMCs. (**A**) Schematic illustrating the genome-wide data sets used to prioritize our search for regulatory elements that control the SMC-selective expression of Prdm6. (**B**) The indicated Prdm6 region was cloned into the appropriate luciferase reporter plasmid and then transfected into HuBrSMCs and mouse ECs. Luciferase activity was measured at 48 hours and is expressed relative to the appropriate empty vector. *n* ≥ 3 for all experimental groups. *P < 0.05 versus promoter less (*t* test); ^#^*P* < 0.05 versus ECs (*t* test). (**C**) DNA sequence of the 325 bp conserved region within the Int3.1 enhancer. (**D**) PCR mutagenesis was used to generate the indicated mutations in the context of the highly active In3.1 conserved regulatory region. Luciferase activity was measured at 48 hours and is expressed relative to the activity of the WT Int3.1 conserved construct. *n* = 5 for all groups. *P < 0.05 versus WT (*t* test). (**E**) Targeted ChIP assays measuring SRF, RBPJ, and TEAD1 binding to the endogenous Int3.1 region. PCR primers used for these experiments are shown in red in **C**. *n* = 3 for all groups. *P < 0.05 versus IgG (*t* test). (**F**) Int3.1 conserved-luciferase was transfected into ECs and SMCs with/without myocardin. Luciferase activity was measured at 48 hours and is expressed relative to Int3.1 conserved luciferase activity in the presence of empty expression vector. *n* = 5 for all groups. *P < 0.05 versus plus empty vector (*t* test). (**G**) PCR mutagenesis was used to generate an allelic series for the rs17149944 variation within the context of Int3.1-luciferase construct. Note that the presence of the minor A allele significantly reduced the activity of the Int3.1 enhancer. *n* = 5 per group. *P < 0.05 versus WT (*t* test).

The fact that noncoding polymorphisms within the Prdm6 gene were associated with both cardiovascular disease and Prdm6 expression in arteries (see Table 1) strongly suggests that proper control of Prdm6 levels is important for normal SMC function. Although Prdm6 exhibits SMC-selective expression in mice (21), and relatively strong expression in human blood vessels (Figure 1D), nothing is known about the mechanisms that drive these expression patterns. To begin to analyze the transcription mechanisms that regulate Prdm6 expression, we took advantage of several genome-wide data sets that we previously generated to characterize chromatin structure and transcription factor binding in HuAoSMCs (16, 19). The Int3.1 region (red box in Figure 9A) was of particular interest for several reasons. It contained a highly conserved 325 bp sequence that included binding motifs for transcription factors known to regulate SMC-specific gene expression (see Figure 9C). It contained a DNase I hypersensitive open chromatin region that was found to be SMC selective when compared to ENCODE data from 7 other non-SMC cell types. It was marked by histone modifications known to be associated with regulatory regions (H3K4 methylation and H3K27 acetylation). It was shown to bind SRF and RBPJ in ChIP-Seq assays. And it contained a genetic variation (rs17149944) that was one of several that define a linkage disequilibrium block associated with blood pressure and Prdm6 expression.

The regulatory regions depicted in green at the bottom of Figure 9A were PCR-amplified from human genomic DNA, subcloned into the appropriate luciferase reporter vectors, and then tested for transcriptional activity in SMCs and endothelial cells (ECs) (to examine cell type specificity). As shown in Figure 9B, the Prdm6 TSS drove high luciferase expression in human bronchial SMCs (HuBrSMCs), but exhibited relatively similar activity in ECs, suggesting that it functions more as a basal promoter. When subcloned upstream of the proximal SV40 promoter, Int2 functioned as a strong repressor, while Int3.2 had little activity. In contrast, the Int3.1 region had significant SMC-selective enhancer activity (~10-fold) within the same context (Figure 9B). An Int3.1 fragment that contained only the 325 bp conserved region exhibited remarkable SMC-selective activity (nearly 70-fold), strongly supporting its role in the SMC-selective expression of Prdm6. Importantly, individual mutations to the consensus SRF, RBPJ, and TEAD binding sites within the conserved Int3.1 region (Figure 9C) significantly inhibited its activity (Figure 9D), and targeted ChIP assays detected binding of each of these factors to the Int3.1 region within the endogenous Prdm6 gene (Figure 9E). Providing additional support for the importance of SRF, overexpression of myocardin significantly increased the activity of the Int3.1 conserved region (Figure 9F).

As shown in Table 1, polymorphisms within an approximately 35 kb region of the Prdm6 third intron (see Figure 9A) have been associated with cardiovascular disease and blood pressure. All 6 are in high linkage disequilibrium (*r*^2^ > 0.78), and the risk allele at each variant has been associated with a similar decrease in Prdm6 expression in human arteries. Thus, any one or more of these could be the causal variant at this important locus. Closer examination of the chromatin environment surrounding these SNPs revealed that only 2 were within regions that might exhibit regulatory activity. Since rs17149944 (but not rs2287696) was within a region that exhibited positive transcriptional activity in luciferase assays (Int3.1), we hypothesized that it was the causal variant and that the minor allele at this locus reduced Prdm6 expression by inhibiting the activity of the Int3.1 enhancer. In support of this idea, mutation of the major rs17149944 allele (G to A) inhibited the activity of the Int3.1 enhancer by over 50% (Figure 9G).

### SMC-specific depletion of Prdm6 in adult mice did not affect BP or the development of hypertension.

**Figure 10:**
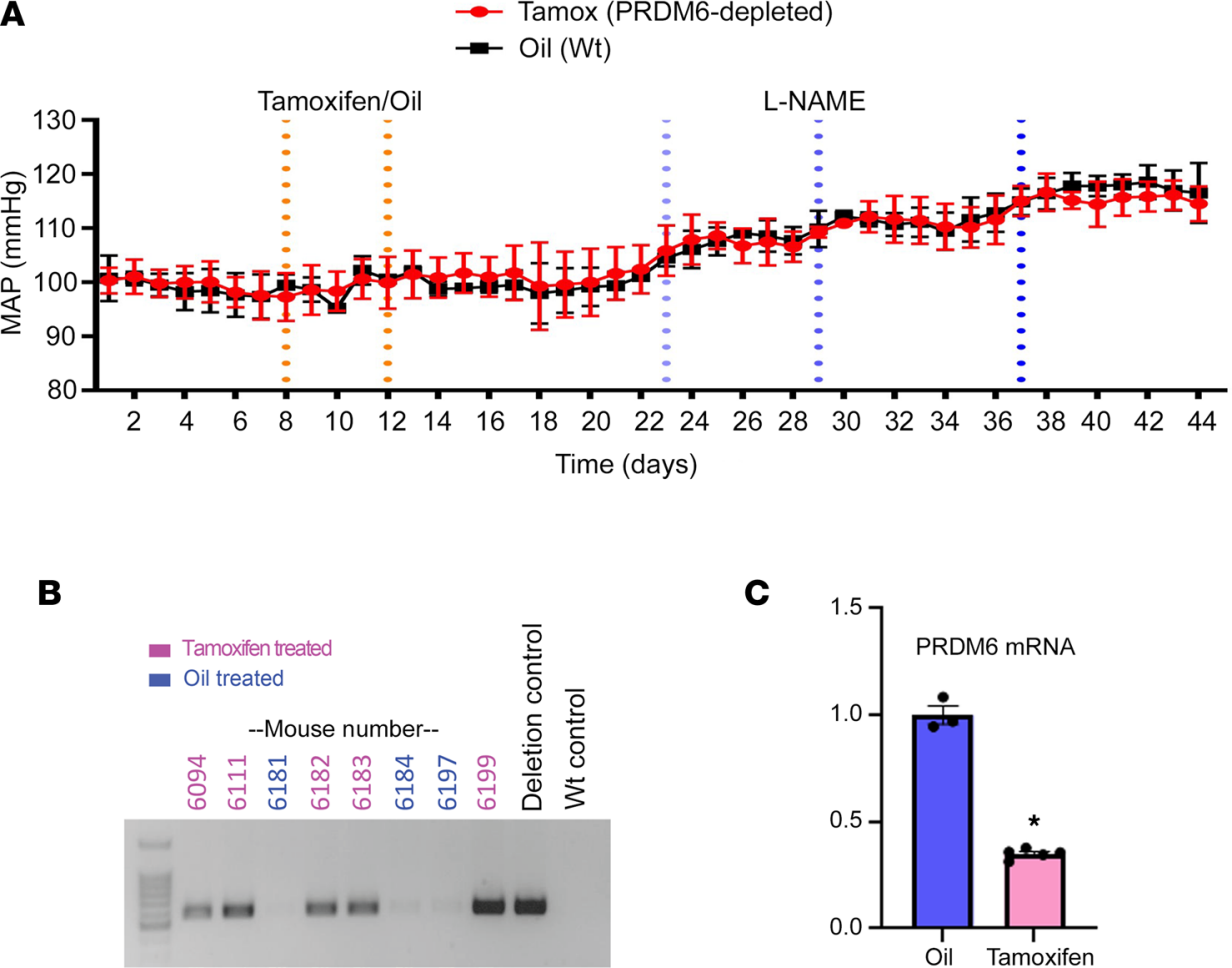
Prdm6 depletion in adult SMCs does not affect blood pressure or l-NAME–induced hypertension. Following telemeter implantation and equilibration, SMMHC^CreERT2^ Prdm6^fl/fl^ mice were treated with 100 mg/kg tamoxifen (*n* = 3) or corn oil (*n* = 3) by oral gavage for 5 consecutive days. l-NAME was added to drinking water at increasing doses (50 mg/L, 150 mg/L, 450 mg/L) 10 days later as indicated. (**A**) Blood pressure (mean arterial pressure, MAP) was monitored continuously by radio telemetry over the entire experiment. All blood pressure measurements are presented as averages over 24-hour periods. (**B**) PCR detection of floxed allele recombination in aorta samples from oil- and tamoxifen-treated SMMHC^CreERT2^ Prdm6^fl/fl^ mice. (**C**) Quantitative PCR–based measurement of *Prdm6* mRNA depletion in aorta samples from oil- and tamoxifen-treated SMMHC^CreERT2^ Prdm6^fl/fl^ mice. *n* = 3–5, *P < 0.05 (*t* test).

The early lethality observed upon deletion of Prdm6 globally or in SM22- or Wnt1-expressing cells has made it difficult to determine whether Prdm6 expression in vascular SMCs plays a role in blood pressure regulation in adult animals. To directly test this, we crossed the Prdm6^fl/fl^ mice with a well-characterized tamoxifen-inducible Cre line driven by the SM MHC promoter (SMMHC^CreERT2^) (44). In brief, following telemeter implantation and equilibration, mice were injected with tamoxifen (or corn oil) for 5 consecutive days, and blood pressure was monitored continuously over the next several weeks by radio telemetry. As shown in Figure 10, we did not detect significant differences between tamoxifen- and vehicle-treated mice, suggesting that Prdm6 depletion does not affect baseline blood pressure. Since many phenotypes in genetically modified mice are only revealed after significant stress, we challenged mice with increasing doses of the NO synthase inhibitor, l-NAME (50 mg/L, 150 mg/L, or 450 mg/L in drinking water), to induce hypertension. Although l-NAME treatment resulted in an 18 mmHg rise in blood pressure, we did not observe significant differences between the tamoxifen- and vehicle-treated groups.

## Discussion

Patent DA is a common congenital condition that frequently afflicts preterm infants. Although human genetic analyses and the reported patent DA phenotypes in many genetically modified mouse models have increased our knowledge of DA function, we do not fully understand the mechanisms that control DA closure. The current studies originally designed to characterize SMC-selective gene expression have led to several interesting insights in this regard. Our data provide strong evidence that Prdm6 expression in DA SMC was required for DA closure, adding support for human genetic data implicating Prdm6 in this process (23). Importantly, during the completion of our studies, Hong et al. also used Wnt1^Cre2^ Prdm6^fl/fl^ mice to demonstrate that Prdm6 depletion in neural crest cells results in a patent DA phenotype (45). Based on lineage tracing results in a Cre-dependent ZsGreen1 reporter mouse line, these authors presented data to suggest that this phenotype was caused by a neural crest migration defect that led to incomplete investment of the ductus SMC layer. As shown in Figure 4, we did not detect differences in neural crest cell investment of the ductus or the morphology of the ductus medial SMC layer. Moreover, as shown (Supplemental Figure 7), PRDM6 depletion in our primary outflow tract SMC culture model failed to affect SMC migration in scratch wound assays. Instead, our data suggest that the patent DA phenotype was mediated by a contractile defect that prevented functional ductus closure, an endpoint not examined by Hong et al. Although the reasons for this discrepancy are unclear, they could involve differences in mouse genetic background, decreased sensitivity of the ZsGreen1 reporter to Cre-based activation (versus ROSA^LacZ^), or differences in the sensitivity of reporter gene detection methods used (i.e., direct detection of ZsGreen1 fluorescence versus Ab-based detection of LacZ). It is important to note that in the same study, these authors reported a patent DA phenotype in SM22^Cre2^ Prdm6^fl/fl^ mice. Since Prdm6 depletion in this model occurs only after the migration and SMC differentiation of neural crest cells, it seems unlikely that the patent DA phenotype observed in Wnt1^Cre2^ Prdm6^fl/fl^ mice was due primarily to a failure of neural crest migration.

Although our RNA-Seq studies support some previous results on DA-selective gene expression (41, 46), they significantly expand the number of genes that define DA identity. The enrichment of the SMC differentiation gene program in the DA strongly suggests that maintenance of SMC differentiation is important for DA function. It is also important to note that many of the genes that were more highly expressed in the DA (versus ascending aorta) were downregulated by Prdm6 depletion; that Prdm6 depletion did not significantly reduce the expression of DA-enriched genes in the aorta, even though Prdm6 was equally expressed in these tissues (see Figure 2C); and that the gene profile observed in Prdm6-deficient DA samples closely resembled that of the WT aorta. Although these data indicate that Prdm6 functions as a critical regulator of DA identity, it is clear that additional signaling mechanisms or transcription factors are important. For example, it is possible that the SMCs that populate the DA and aorta originate from slightly different neural crest cell populations and thus express a different array of developmental transcription factors. The Gittenberger-de Groot group has suggested that homeobox b5 (Hoxb5) may be important for ductus identity because it is selectively expressed in the sixth pharyngeal arch, which eventually remodels into the DA (47). Although we did detect more Hoxb5 expression in the DA than in the ascending aorta by RNA-Seq, Hoxb5 levels were not altered by PRDM6 depletion. Nevertheless, it will be important to test whether Hoxb5 and Prdm6 interact functionally. The regulation of SMC phenotype is also highly dependent upon local environmental cues, including those that sense mechanical forces (48). Given that the DA is exposed to very different mechanical signals from the rest of the outflow tract, it is possible that this leads to DA-selective changes in gene expression that modify the baseline identity of neural crest–derived SMCs.

The dramatic effect of Prdm6 depletion on myogenic tone and contractility of isolated DA segments provides convincing evidence that Prdm6 is required for normal DA constriction. However, since Prdm6 depletion in DA SMCs resulted in many significant gene expression changes, it is difficult to ascertain which mediate the contractile deficiencies observed. The downregulation of the SMC gene program may be important, and the fact that patent DA phenotypes have been observed in neural crest cell–specific myocardin-KO (49) and global SMMHC-KO mice (50) adds support for this idea. However, even though Prdm6 depletion decreased SMC differentiation marker gene expression, mRNA levels for the SMC markers remained relatively high compared with non-SMC cell types and were not much different than that observed in ascending aorta samples from WT mice. EP4 expression in the DA was decreased by 90% in PRDM6-deficient DA samples. Although this receptor is highly and selectively expressed in the DA (see Figure 8) and is known to mediate DA relaxation, its knockout leads to a paradoxical patent DA phenotype (51), suggesting that DA closure is regulated by additional pathways and that compensatory mechanisms may be activated when DA closure signals are altered. The DA-selective transcription factor, Tfapb2, was also dramatically downregulated by more than 90%, and a critical next step will be to test whether restoring the expression of this, or other candidate genes, can rescue the effects of Prdm6 depletion.

Although many of the genes altered by Prdm6 depletion were also shown to bind Prdm6 by ChIP-Seq, the mechanisms by which Prdm6 alters gene expression are still unclear. The present studies were initiated by our demonstration that Prdm6 could bind MRTF-A, and the observation that Prdm6 depletion decreased SMC marker gene expression supports the idea that these factors cooperate to regulate SMC identity. However, with a few exceptions, we did not detect Prdm6 binding at the SMC-specific promoters, perhaps suggesting that the effects of Prdm6 on SMC identify may be secondary to effects on other transcription mechanisms. We did detect Prdm6 binding to a region shown to drive SMC-selective expression of myocardin (52), and it will be interesting to test whether Prdm6 interacts with the Mef2c, TEAD, and Fox transcription factors that mediate the regulatory activity of this enhancer. The fact that consensus cis elements for the TEAD and AP1 family of transcription factors were overrepresented in our Prdm6 ChIP-Seq data set suggests that Prdm6 may interact with additional transcription factors to control gene expression in SMCs. It is also possible that PRDM6 helps recruit MRTF-A to the promoters of important ductus genes that are not necessarily SMC selective or SRF dependent.

Many GWAS have identified genetic loci that contribute to variations in blood pressure and other cardiovascular disease endpoints. However, most of these are within or near genes that have no known connection to cardiovascular function and are defined by multiple noncoding variations in high linkage disequilibrium. Thus, critical next steps are to identify the causal variants at these loci and to determine the mechanism by which they exert their effects. The current studies are the first to our knowledge to identify the transcription mechanisms that control Prdm6 expression in SMCs, and our characterization of an SMC-selective enhancer within the Prdm6 third intron is an important advance. The observation that Int3.1 activity is mediated by SRF, RBPJ, and TEAD, 3 transcription factors known to be important for SMC-specific gene expression (17, 53, 54), suggests that the activation of Prdm6 expression in outflow tract SMCs during development is likely downstream or concurrent with SMC specification. Providing some evidence for this possibility, Li et al. have shown that MRTF-B is expressed in premigratory neural crest cells at E8.5 (several days before the activation of Prdm6) and that MRTF-B–deficient mice have defects in neural crest SMC differentiation and outflow tract development (55). The observations that PRDM6 expression was required for full myocardin expression and that the Int3.1 conserved region was transactivated by myocardin suggests a potential positive feedback loop that could help drive strong SMC identity. Our data also provide evidence that rs17149944 is the causal SNP for the cardiovascular disease locus within the Prdm6 third intron, though it is difficult to completely rule out the involvement of other variants. Based upon ENCODE data, rs17149944 is located within a CTCF ChIP-Seq peak detected in 84 separate cell lines just 3 bp downstream of a consensus CTCF binding sequence. Thus, it may exert its effects on Prdm6 expression by altering chromatin looping between the Int3.1 enhancer and the Prdm6 TSS. Of interest, in addition to being in high linkage disequilibrium with 3 variants directly implicated in blood pressure control, rs10077410, rs2287696, and rs13359291 (24, 26–28), the rs17174994 variant itself has been associated with atrial fibrillation (29). Given that the vagus nerve develops from the fourth pharyngeal arch (56) and that Prdm6 depletion increased neural gene expression in our study, it is possible that altered Prdm6 expression in neural crest–derived cells contributes to this association.

Somewhat surprisingly, we did not observe effects on blood pressure when Prdm6 expression was depleted in adult SMCs. Compensation by one or more of the many diverse mechanisms that maintain blood pressure homeostasis may have masked an effect of Prdm6 in our model, but we cannot rule out that the effects of Prdm6 on blood pressure are mediated by as-yet-uncharacterized expression in a non-SMC cell type. The association of Prdm6 with blood pressure could also be due to effects of Prdm6 during development. Given our demonstration that Prdm6 affected DA contractility, it is possible that similar effects in the vascular tree could lead to subtle structural alterations or compensatory remodeling that have downstream effects on blood pressure regulation in adults. Of interest in this regard, the third intron locus that contains rs17149944 has also been associated with pulse pressure (28), an endpoint usually mediated by increased aortic stiffening.

In summary, our studies provide potentially novel evidence for the importance of Prdm6 in DA function and closure. Our data suggest that Prdm6 is critical for a DA-selective gene program characterized in part by strong expression of the SMC differentiation marker genes. We show for the first time to our knowledge that Prdm6 depletion in neural crest–derived SMCs results in decreased DA tone and contractility, likely explaining the patent DA phenotype observed in Wnt1^Cre2^ Prdm6^fl/fl^ mice and in humans that have Prdm6-coding mutations. Our characterization of the regulatory elements that drive the SMC-selective expression of Prdm6 should add to our understanding of DA development and the mechanisms by which cardiovascular disease–associated variations within the Prdm6 affect its expression. Finally, our studies provide a strong foundation for future examination of mechanisms that control DA contractility and closure.

## Methods

### Animal models.

All mouse lines examined were on a C57/Black6 background (at least 6 backcrosses). To knock out Prdm6 in neural crest cells, Prdm6^fl/fl^ mice (a gift from Jurgen Ruland, Technical University of Munich, Munich, Germany) (22) were crossed with the well-characterized Wnt1^Cre2^ transgenic line (The Jackson Laboratory) (33) in which Cre-recombinase is driven by the Wnt1 promoter. Wnt1^Cre2^ Prdm6^fl/fl^ mice were generated by crossing male and female Wnt1^Cre2^ Prdm6^wt/fl^ mice and were analyzed at several developmental time points, including E13.5, E15.5, E17.5, E18.5, P1, and P2. It is important to note that Wnt1^Cre2^ Prdm6^wt/fl^, Wnt1^Cre2^ Prdm6^wt/wt^, and Prdm6^fl/fl^ mice were phenotypically indistinguishable from C57/Black6 mice and were used as littermate controls in all experiments. For lineage tracing Wnt1^Cre2^ Prdm6^fl/fl^ mice were bred to the ROSA26^LacZ^ indicator strain (The Jackson Laboratory) (44). To knock out Prdm6 in adult SMCs, Prdm6^fl/fl^ mice were crossed with a tamoxifen-inducible Cre line driven by the SM MHC promoter (SMMHC^CreERT2^) (from Stefan Offermanns, Max Planck Institute, Bad Nauheim, Germany) (44). Cre activity in this model was activated by treatment with tamoxifen (100 mg/kg) by oral gavage for 5 consecutive days.

### LacZ tissue staining.

Wnt1^Cre2^ Prdm6^fl/fl^ ROSA26^LacZ^ and Wnt1^Cre2^ ROSA26^LacZ^ mice were euthanized at P1, and chest cavities were opened to expose the heart and outflow tract. After a brief rinse in phosphate-buffered saline (PBS), mice were fixed in 4% paraformaldehyde (PFA) for 20 minutes. After three 10-minute incubations in wash buffer (0.1 M phosphate buffer pH 7.3, 0.1% sodium deoxycholate, 0.02% NP-40, 0.05% BSA), pups were incubated overnight at room temperature in X-gal staining solution (wash buffer containing 1 mg/mL X-gal from MilliporeSigma, 5 mM ferrocyanide, and 5 mM ferricyanide) as previously described (18).

### Histology, immunofluorescence, and in situ hybridization.

Mouse embryos collected by C-section were rinsed in PBS. After removing the head and neck, embryos were either snap-frozen in Tissue-Tek OCT compound (VWR 102094-106) and stored at –80°C or fixed in 4% PFA for 24 hours and processed for paraffin-embedding. Both frozen and paraffin-embedded embryos were cut into 10- to 15-micron sections, and those that contained the outflow tract arteries were collected onto charged slides. For histological observations, sections were stained with H&E using standard protocols. For immunostaining, frozen sections were thawed and then fixed in ice-cold acetone for 10 minutes while paraffin-embedded sections were deparaffinized, rehydrated, and subjected to antigen retrieval by incubating in 10 mM sodium citrate buffer (pH 6.0) at 95°C for 10 minutes. All sections were then washed 3 times in 1× PBS and blocked for 1 hour at room temperature with 5% goat serum in PBS. Abs against the following proteins were applied overnight at 4°C; β-galactosidase (Thermo Fisher Scientific PA5-102503, 1:100), CNN1 (Abcam ab46794, 1:250), CD31 (Abcam ab56299, 1:400), and SM α-actin (MilliporeSigma A5228). Sections were washed with PBS 3 times for 10 minutes, then incubated with secondary Abs (A21202 and A21422 from Invitrogen) (1:500) and DAPI (1:1,000) for 1 hour at room temperature before being mounted with Fluoromount-G mounting medium (SouthernBiotech 0100-01). For RNAscope-based in situ hybridization, slides were processed and stained using the RNAscope Multiplex Fluorescent V2 Assay (ACDBio 323110) according to manufacturer instructions. Target retrieval was performed at 99°C for 15 minutes, followed by Protease Plus (ACDBio 322331) treatment at 40°C for 30 minutes. Probes used include Prdm6 (ACDBio 456891), Tfap2b (ACDBio 536371-C2), and EP4 (ACDBio 441461-C3). Images were taken with a confocal microscope (Zeiss LSM 700 Confocal Laser Scanning Microscope) and were analyzed using ImageJ/Fiji software.

To better visualize the status of the DA in mice, intracardiac perfusion was performed using Microfil silicone rubber injection compound (MV-122; Flow Tech Inc.) per manufacturer’s instructions.

### Indomethacin treatment.

A single dose of indomethacin (20 mg/kg) was administered to pregnant dams at E18.5 by oral gavage. Four hours after dosing, fetuses were delivered by C-section and processed for histological analysis as described above.

### RNA-Seq.

E18.5 embryos were collected from Wnt1^Cre2^ Prdm6^fl/fl^ (*n* = 5) and littermate control mice (*n* = 7). Ascending aorta and DA samples were isolated by microdissection, and total RNA from each sample was isolated using the RNeasy UCP Micro Kit (QIAGEN 73934) per manufacturer’s instructions. Low-input RNA-Seq (151 bp, paired-end) was performed by Novogene Corporation Inc., with an average of 25.8 million paired reads generated per sample. All RNA samples sequenced had RNA integrity number values greater than 9.0. For each sample, sequences were filtered using fastq quality filter (FASTX-Toolkit) (RRID:SCR_005534) requiring at least 90% of bases (-p 90) to have a minimum quality score of 20 (-q 20). Adapter sequences were removed using tagdust (57) at a false discovery rate (-f) of 0.001. Sequences were aligned to the mm10 mouse reference genome and GENCODE (58) version 20 gene annotation with STAR (59) using default parameters, with an average of almost 95% of reads aligned. Expression was quantified with rsem (60) using default parameters. Data were deposited in the National Center for Biotechnology Information’s Gene Expression Omnibus (NCBI GEO), accession number GSE221004.

### Pressurized vessel myography.

Ductus and ascending aorta segments from 4 to 8 fetuses representing at least 4 litters were used for each myography study. Wnt1^Cre2^ Prdm6^fl/fl^ and control vessels were freshly isolated, and vasoreactivity was evaluated using cannulated, pressurized vessel myography and computer-assisted videomicroscopy, as previously described (38–41, 61). Briefly, the excised vessel was mounted in custom myography chambers (University of Vermont), then equilibrated for 40 minutes at 37°C and 5 mmHg of distending pressure while submerged in modified, deoxygenated Krebs buffer. Chambers were placed on an inverted microscope equipped with a digital image capture system (IonOptix) to record changes in intraluminal diameter. Pressure was increased to 20 mmHg in 5 mmHg increments followed by exposure to deoxy buffer containing 50 mM KCl (in mM: 64 NaCl, 50 KCl, 2.5 CaCl_2_ 2H_2_O, 0.9 MgSO_4_, 1 KH_2_PO_4_, 11.1 glucose, 34 NaHCO_3_, pH 7.3) to determine vessel viability and peak contractility. Chambers were then changed from a flow-through system to a recirculating system (20 mL total volume) and allowed to re-equilibrate for 20 minutes. Lumen diameter was initially recorded as the resting diameter at 5 mmHg baseline for deoxygenated conditions. Pressure-induced tone (myogenic response) was monitored as the change in lumen diameter (from peak to trough after 10 minutes at each new pressure) during the pressure ramps. In some studies, progressive changes in lumen diameter in response to increased concentrations of KCl (12.5, 25, 50 mM) were recorded and compared. For oxygen treatment studies, vessels were changed from a recirculating system that was continuously aerated with deoxygenated gas (pO_2_ ~38–42 Torr) to one aerated with increasing concentrations of oxygen (Krebs buffer bubbled with either 0, 2, 5, 12, 21, or 95% O_2_/5% CO_2_/balanced N_2_) (38) until a new lumen diameter was recorded. Before each increase in oxygen concentration, lumen diameters were allowed approximately 10 minutes to achieve a new stable baseline (maximum of 20 minutes). Under 95% oxygen conditions, vessels were exposed to the thromboxane receptor agonist U46619 (10^–7^ M; MilliporeSigma) to evaluate maximal contractile response. At the end of each study, vessels were exposed to 50 mM KCl to verify vessel responsiveness and integrity.

### Blood pressure measurements.

Radiotelemetry (Data Sciences International) was used to measure blood pressure in tamoxifen- and vehicle-treated SMMHC^CreERT2^ Prdm6^fl/fl^ mice aged 12–16 weeks as previously described (18). In some experiments, l-NAME was added to drinking water at increasing doses (50 mg/L, 150 mg/L, 450 mg/L) every 7 days.

### Cell culture, transfections, and luciferase assays.

HuBrSMCs were purchased from Lonza and maintained in Clonetics Smooth Muscle Growth Medium-2. Primary rat aortic SMCs were isolated as previously described (62) and maintained in DMEM:F12 supplemented with 10% FBS and 0.5% penicillin/streptomycin. Outflow tract SMCs were isolated from the aortic arch and the DA of Prdm6^fl/fl^ mouse embryos at E18.5 as previously described (63). After 3 passages, more than 95% of cells were positive for SM α-actin and calponin-1 and showed a typical elongated cell morphology. Immortalized primary mouse ECs were previously described (64). COS-7 cells (ATCC), multipotential 10T1/2 cells (ATCC), and ECs (64) were maintained in DMEM supplemented with 10% FBS and 0.5% penicillin/streptomycin. Cells were transfected the day after plating at 70 to 80% confluence with the Transit-LT1 reagent (Mirus) per manufacturer’s instructions. Prdm6 regulatory elements were PCR-amplified from HuBrSMC genomic DNA and subcloned into pGL3 basic or pGL3 promoter vectors (Promega). Site-directed mutations were generated by the QuickChange protocol (Agilent Technologies) and were verified by Sanger sequencing. For luciferase assays, cells were seeded in 48-well plates at a density of 2.5 × 10^4^ cells/well and transfected with 250 ng of luciferase plasmid per well. Luciferase activity was measured 48 hours after transfection using the Steady-Glo Luciferase Kit (Promega) according to the manufacturer’s instructions. Raw luciferase values were normalized to the activity of the empty pGL3 basic or pGL3 promoter vectors. For transactivation experiments, 125 ng of expression plasmids (empty vector or myocardin) was cotransfected along with 125 ng of luciferase plasmid.

### siRNA knockdowns.

Cells were transfected with 30 nM siRNA targeting Prdm6 or GFP using Dharmafect transfection reagent (Dharmacon). Quantitative PCR for Prdm6 and several SMC markers was performed 72 hours after transfection. siRNA sequences used were Prdm6 5′GGUGCGCUCCUGGACGUAGCC3′ and GFP 5′GGUGCGCUCCUGGACGUAGCC3′.

### ChIP assays.

ChIP assays were performed as previously described (16) according to the SimpleChIP protocol (Cell Signaling Technology). Following chromatin digestion and shearing, immunoprecipitation was performed with 1 to 5 μg of one of the following Abs overnight at 4°C: anti-RBPJ (Cell Signaling Technology; catalog D10A4), anti-TEAD1 (Santa Cruz Biotechnology; catalog 393976), anti-SRF (Santa Cruz Biotechnology; catalog sc-335), anti-flag (Cell Signaling Technology; catalog 8146S), nonimmune rabbit IgG (Cell Signaling Technology; catalog 2729), or nonimmune mouse IgG (MilliporeSigma; catalog NI03). For PRDM6 ChIP-Seq experiments, flag-Prdm6 was subcloned into the p-Lenti vector (Addgene), then cotransfected into HEK293T cells (ATCC) with packaging vectors psPAX2 (Addgene plasmid 12260) and pMD2.G (Addgene plasmid 12259). Lentiviral particles were purified using standard protocols and then used to treat outflow tract SMCs. ChIP-Seq assays were performed on 2 independent replicates using an anti-flag Ab (Cell Signaling Technology; catalog 14793). Input control libraries were generated for each replicate, in which all steps except the immunoprecipitation were performed. Sequencing (151 bp, paired-end) of resulting DNA libraries was performed by Novogene Corporation Inc. Reads with >50% low-quality bases (<20) and with >15% uncalled bases (N) were removed. Adapters were trimmed and resulting reads <18 bases were removed. The remaining sequences were aligned to the mm10 reference genome using BWA (65) requiring a mapping quality of at least 13. Initial peaks were called using MACS2 (66) using matching input controls (*P* < 0.001). Overrepresented sequences in the ChIP-Seq data set were identified using HOMER (67). Data were deposited in the NCBI GEO, accession number GSE221094.

### Immunoprecipitations and Western blotting.

Epitope-tagged Prdm6 and MRTF-A expression constructs were transfected into COS-7 or 10T/12 cells. After 48 hours cells were lysed in RIPA buffer plus protease and phosphatase inhibitors. Approximately 1 mg of protein was incubated with 3 to 5 μg of anti-flag (Cell Signaling Technology; catalog 8146S), anti-myc (Cell Signaling Technology; catalog 2276s), or nonimmune mouse IgG (MilliporeSigma; catalog NI03) overnight at 4°C with rotation. Immunoprecipitants were washed 3 times in wash buffer (20 mM Tris HCl, pH7.3, 150 mM NaCl, 10% glycerol, 0.5% Triton X-100), eluted in sample buffer, resolved by SDS-PAGE, transferred to nitrocellulose, and probed with anti-flag (Cell Signaling Technology; catalog 8146S), anti-myc (Cell Signaling Technology; catalog 2276s), or anti-MRTF-A (sc-32909, Santa Cruz Biotechnology) Abs. For Western blots used to measure SMC differentiation marker gene expression, cleared RIPA lysates were boiled in sample buffer, run on an 10% SDS-PAGE gel, transferred to nitrocellulose, and probed with the following Abs: SM α-actin (MilliporeSigma, catalog A5228), CNN1 (LifeSpan Bio catalog LS-B7497), SM22 (Santa Cruz Biotechnology; catalog sc-271719), and GAPDH (Cell Signaling Technology; catalog 97166s).

### Far Western.

Flag-tagged MRTF-A variants were immunoprecipitated from COS-7 cells as above, resolved by SDS-PAGE, transferred to nitrocellulose, and then renatured according to previously established protocols (68). Blots were then incubated with recombinant GST-PRDM6 protein diluted in blocking buffer (20% goat serum, 3% BSA, in PBS) and then probed with an anti-GST Ab (Cell Signaling Technology; catalog 2622s).

### Liquid chromatography-tandem mass spectrometry analysis.

An anti–MRTF-A Ab (sc-32909, Santa Cruz Biotechnology) was used as above to immunoprecipitate endogenous MRTF-A from mouse AoSMCs. Washed immunoprecipitates were submitted directly for analysis by mass spectroscopy on a nanoACQUITY-Orbitrap Velos system. Samples were eluted over a 150-minute gradient from 1% to 40%, where mobile phase A was 0.1% formic acid and mobile phase B was acetonitrile with 0.1% formic acid. The top 8 most intense ions were chosen for higher energy collisional dissociation. Peptide coverage of immunoprecipitated MRTF-A was 26.8%.

### Statistics.

All data represent at least 3 independent experiments presented as means ± SEM. Means were compared by Student’s 2-tailed *t* test, and statistical significance was considered as a *P* value less than 0.05. For myography studies, change in lumen diameter was plotted as percentage change compared with baseline diameter at 20 mmHg resting tone (Prism 6, GraphPad Software). One-way ANOVA with repeated measures was used to detect differences in vessel caliber in response to increasing concentrations of KCl or oxygen. Knockout and control vessel response curves were compared by 2-way ANOVA. Bonferroni’s post hoc analysis was performed when significant differences were found. An unpaired 2-tailed Student’s *t* test was used to compare the effects of the thromboxane agonist, U46619, on DA and aorta contractility.

### Study approval.

All animal procedures were approved by the University of North Carolina and/or Vanderbilt University Institutional Animal Care and Use Committees. All animals were housed in facilities accredited by the American Association for Accreditation of Laboratory Animal Care.

## Author contributions

MZ designed research studies, conducted experiments, collected data, analyzed data, and wrote the manuscript. KDM designed research studies, conducted experiments, collected data, analyzed data, and wrote the manuscript. JCM designed research studies, conducted experiments, collected data, and analyzed data. HHC conducted experiments, collected data, and analyzed data. MTY conducted experiments, collected data, and analyzed data. ELS designed research studies, conducted experiments, collected data, analyzed data, and provided reagents. JMT designed research studies, analyzed data, wrote the manuscript, and provided reagents. JR designed research studies, conducted experiments, collected data, analyzed data, and provided reagents. TSF designed research studies, analyzed data, wrote the manuscript, and provided reagents. CPM designed research studies, conducted experiments, collected data, analyzed data, wrote the manuscript, and provided reagents.

## Supplementary Material

Supplemental data

## Figures and Tables

**Table 1:**
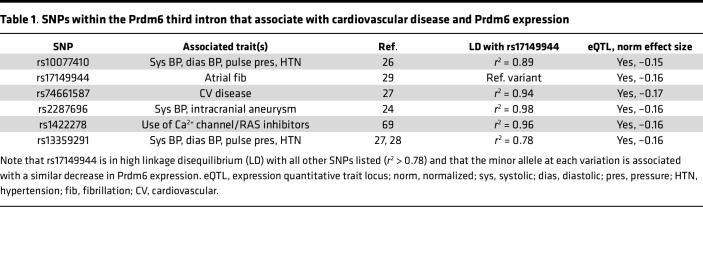
SNPs within the Prdm6 third intron that associate with cardiovascular disease and Prdm6 expression

## Version 1 02/07/2023

In-Press Preview

## Version 2 03/08/2023

Electronic publication
