# Supplementary material for: Prdm6 drives ductus arteriosus closure by promoting ductus arteriosus smooth muscle cell identity and contractility
Source: JCI Insight. 2023 Mar 8;8(5):e163454. doi: 10.1172/jci.insight.163454 (PMC10077476; doi:10.1172/jci.insight.163454)
Supplement: Supplemental data [file jciinsight-8-163454-s069.pdf]

Supplemental Table I. MRTF-A binding partners in mouse SMC. Endogenous MRTF-A was immunoprecipitated from mouse AoSMC lysates, and binding partners were identified by LS/MS/MS analysis.

Uniprot Accession	Name	Abbreviation
Q5SWU9	acetyl-Coenzyme A carboxylase alpha	Acaca
Q07417	acyl-Coenzyme A dehydrogenase, short chain	Acads
Q8K3H0	adaptor protein, phosphotyrosine interaction, PH domain and leucine zipper containing 1	Appl1
A2AE38	adhesion molecule with Ig like domain 1	Amigo1
O89020	afamin	Afm
P24549	aldehyde dehydrogenase family 1, subfamily A	Aldh1a1
A6ZI44	aldolase A, fructose-bisphosphate	Aldoa
D3YW52	alpha-2-macroglobulin	A2m
F8VPN4	amylo-1,6-glucosidase, 4-alpha-glucanotransferase	Agl
E9Q414	apolipoprotein B	Apob
P48999	arachidonate 5-lipoxygenase	Alox5
P61164	ARP1 actin-related protein 1A, centractin alpha	Actr1a
P41233	ATP-binding cassette, sub-family A (ABC1), member 1	Abca1
Q8K440	ATP-binding cassette, sub-family A (ABC1), member 8b	Abca8b
Q9JJ59	ATP-binding cassette, sub-family B (MDR/TAP), member 9	Abcb9
Q9WU60	attractin	Atrn
Q9JLV1	BCL2-associated athanogene 3	Bag3
P59017	BCL2-like 13 (apoptosis facilitator)	Bcl2l13
Q8CI94	brain glycogen phosphorylase	Pygb
Q6GQW0	BTB (POZ) domain containing 11	Btbd11
A0PJK7	cadherin-related family member 5	Cdhr5
Q923T9	calcium/calmodulin-dependent protein kinase II gamma	Camk2g
A0A0G2JGS4	calcium/calmodulin-dependent protein kinase II, delta	Camk2d
O88456	calpain, small subunit 1	Capns1
Q08093	calponin 2	Cnn2
P47936	cannabinoid receptor 2 (macrophage)	Cnr2
B2RS76	carboxypeptidase B1 (tissue)	Cpb1
Q60737	casein kinase 2, alpha 1 polypeptide	Csnk2a1
Q549Q4	CD2 antigen	Cd2
Q3TI84	CDC16 cell division cycle 16	Cdc16
E9PVY0	CDC42 binding protein kinase alpha	Cdc42bpa
Q7TT50	CDC42 binding protein kinase beta	Cdc42bpb
Q6NV72	cDNA sequence BC068281	BC068281
Q8CH18	cell division cycle and apoptosis regulator 1	Ccar1
Q6A065	centrosomal protein 170	Cep170
Q99LI7	cleavage stimulation factor, 3' pre-RNA, subunit 3	Cstf3
D3YUP1	coactivator-associated arginine methyltransferase 1	Carm1
Q02788	collagen, type VI, alpha 2	Col6a2
P01027	complement component 3	C3
P01029	complement component 4B (Chido blood group)	C4b
A2A432	cullin 4B	Cul4b
Q8CID0	cysteine and glycine-rich protein 2 binding protein	Csrp2bp
Q7TMB8	cytoplasmic FMR1 interacting protein 1	Cyfip1
K3W4R0	dynein, axonemal, heavy chain 17	Dnah17
L7N1Y0	dynein, axonemal, heavy chain 7B	Dnah7b
B2RWS6	E1A binding protein p300	Ep300
Q8BL66	early endosome antigen 1	Eea1
Q05BC3	echinoderm microtubule associated protein like 1	Eml1
F8WJ93	echinoderm microtubule associated protein like 4	Eml4
E9QAU4	enhancer trap locus 4	Et14
Q8BH95	enoyl Coenzyme A hydratase, short chain, 1, mitochondrial	Echs1
Q8BGS1	erythrocyte membrane protein band 4.1 like 5	Epb41l5
Q3ULL5	eukaryotic translation initiation factor 2, subunit 2 (beta)	Eif2s2
Q3UW53	family with sequence similarity 129, member A	Fam129a
Q3UW64	glucosamine (UDP-N-acetyl)-2-epimerase/N-acetylmannosamine kinase	Gne
Q9CQZ1	heat shock factor binding protein 1	Hsbp1
Q9JK92	heat shock protein 8	Hspb8
P01942	hemoglobin alpha, adult chain 1	Hba-a1
Q3UDW8	heparan-alpha-glucosaminide N-acetyltransferase	Hgsnat
Q20BD0	heterogeneous nuclear ribonucleoprotein A/B	Hnrnpab
Q8VDM6	heterogeneous nuclear ribonucleoprotein U-like 1	Hnrnpul1
Q9JIY5	HtrA serine peptidase 2	Htra2
O88703	hyperpolarization-activated, cyclic nucleotide-gated K+ 2	Hcn2
P01751	immunoglobulin heavy variable 1-72	Ighv1-72
A0A075B666	immunoglobulin kappa chain variable 13-85	Igkv13-85
Q91YE6	importin 9	1po9
D3Z627	integrin alpha L	Itgal
Q9QXH4	integrin alpha X	Itgax
E9QAD8	IQ motif and Sec7 domain 2	Iqsec2
E9Q9B7	kinase D-interacting substrate 220	Kidins220)
E9Q0J5	kinesin family member 21A	Kif21a
B7ZNG0	kinesin family member 7	Kif7
Q91W40	kinesin light chain 3	Klc3

Supplemental Table I. (continued)

B2RWI2	lactamase, beta	Lactb
Q8R502	leucine rich repeat containing 8 family, member C	Lrrc8c
Q8CGK3	lon peptidase 1, mitochondrial	Lonp1
V9GX48	M-phase phosphoprotein 9	Mphosph9
Q9EQQ9	meningioma expressed antigen 5 (hyaluronidase)	Mgea5
P25206	minichromosome maintenance complex component 3	Mcm3
Q52KC3	minichromosome maintenance complex component 5	Mcm5
Q80X85	mitchondrial ribosomal protein S7	Mrps7
Q8K4J6	MKL (megakaryoblastic leukemia)/myocardin-like 1	Mkl1
Q3U2W2	MYB binding protein (P160) 1a	Mybbp1a
Q80TM9	nischarin	Nisch
Q99K48	non-POU-domain-containing, octamer binding protein	Nono
Q60632	nuclear receptor subfamily 2, group F, member 1	Nr2f1
Q9CQF3	nudix (nucleoside diphosphate linked moiety X)-type motif 21	Nudt21
Q8CGY8	O-linked N-acetylglucosamine (GlcNAc) transferase (UDP-N-acetylglucosamine:polypeptide-N-acetylglucosaminyl transferase)	Ogt
Q8VGB4	olfactory receptor 985	Olfr985
A2AEG2	oral-facial-digital syndrome 1 gene homolog (human)	Ofd1
B2RRE7	OTU domain containing 4	Otud4
A0A0G2JDJ3	paired immunoglobin-like type 2 receptor alpha	Pilra
Q9R0L6	pericentriolar material 1	Pcm1
Q8BVZ1	perilipin 5	Plin5
Q8BH04	phosphoenolpyruvate carboxykinase 2 (mitochondrial)	Pck2
Q64737	phosphoribosylglycinamide formyltransferase	Gart
Q8K1N2	pleckstrin homology like domain, family B, member 2	Phldb2
B2RXS4	plexin B2	Plxnb2
P59470	polymerase (RNA) III (DNA directed) polypeptide B	Polr3b
Q3UZD5	PR domain containing 6	Prdm6
Q3V0P3	predicted gene 1527	Gm1527
Q58EV5	predicted gene, 21596	Gm21596
P70268	protein kinase N1	Pkn1
B2RXQ2	protein tyrosine phosphatase, receptor type, f polypeptide (PTPRF), interacting protein (liprin), alpha 1	Ppfia1
Q3TMZ1	pyrroline-5-carboxylate reductase family, member 2	Pycr2
Q9DCC4	pyrroline-5-carboxylate reductase-like	Pycrl
O35551	rabaptin, RAB GTPase binding effector protein 1	Rabep1
Q3UYI5	ral guanine nucleotide dissociation stimulator-like 3	Rgl3
E9PW37	RAS protein activator like 2	Rasal2
Q5SWN2	replication protein A1	Rpa1
Q99P72	reticulon 4	Rtn4
Q9EP71	retinoic acid induced 14	Rai14
E9PUF7	Rho guanine nucleotide exchange factor (GEF) 1	Arhgef1
Q8C033	Rho guanine nucleotide exchange factor (GEF) 10	Arhgef10
F8VQN6	Rho guanine nucleotide exchange factor (GEF) 12	Arhgef12
Q91VI7	ribonuclease/angiogenin inhibitor 1	Rnh1
P62918	ribosomal protein L8	Rpl8
P62855	ribosomal protein S26	Rps26
A0A087WRF9	RIKEN cDNA 1700088E04 gene	1700088E04Rik
E9QM90	RIKEN cDNA 2310035C23 gene	2310035C23Rik
G3X8V5	ring finger protein 219	Rnf219
F8WJE0	SAM domain and HD domain, 1	Samhd1
Q9EQC5	SCY1-like 1 (S. cerevisiae)	Scyl1
A2AIX1	SEC16 homolog A, endoplasmic reticulum export factor	Sec16a
M9MMK0	sema domain, immunoglobulin domain (Ig), short basic domain, secreted, (semaphorin) 3B	Sema3b
Q543J5	serine (or cysteine) peptidase inhibitor, clade C (antithrombin), member 1	Serpinc1
Q9JI11	serine/threonine kinase 4	Stk4
Q6ZPE2	SET binding factor 1	Sbf1
Q3TRJ7	SH3-domain GRB2-like 1	Sh3gl1
P56873	Sjogren's syndrome/scleroderma autoantigen 1 homolog (human)	Sssca1
F8VPQ4	SLIT-ROBO Rho GTPase activating protein 3	Srgap3
Q6P5D8	SMC hinge domain containing 1	Smchd1
Q80UJ1	solute carrier family 22 (organic anion transporter), member 20	Slc22a20
Q58A65	sperm associated antigen 9	Spag9
Q8VIJ6	splicing factor proline/glutamine rich (polypyrimidine tract binding protein associated)	Sfpq
O54988	STE20-like kinase	Slk
P58871	tankyrase 1 binding protein 1	Tnks1bp1
Q9D2E2	target of EGR1, member 1 (nuclear)	Toe1
Q3URV1	TBC1 domain family, member 32	Tbc1d32
Q05895	thrombospondin 3	Thbs3
A2ASS6	titin	Ttn
Q52L67	trans-2,3-enoyl-CoA reductase	Tecr
Q62351	transferrin receptor	Tfrc
P37804	transgelin	Tagln
Q3UBX0	transmembrane protein 109	Tmem109
Q80WC3	trinucleotide repeat containing 18	Tnrc18
Q9Z1A1	Trk-fused gene	Tfg
Q6F4J0	tubulin, gamma 2	Tubg2
Q9ES34	ubiquitin protein ligase E3B	Ube3b
F6WJB7	ubiquitin specific peptidase 34	Usp34
F8VPU6	ubiquitin specific peptidase 9, Y chromosome	Usp9y
Q9CR26	vesicle (multivesicular body) trafficking 1	Vta1
E9Q743	WD repeat domain 66	Wdr66
Q6NXJ0	WW, C2 and coiled-coil domain containing 2	Wwc2
Q9JKB3	Y box protein 3	Ybx3

Suppl Fig 1

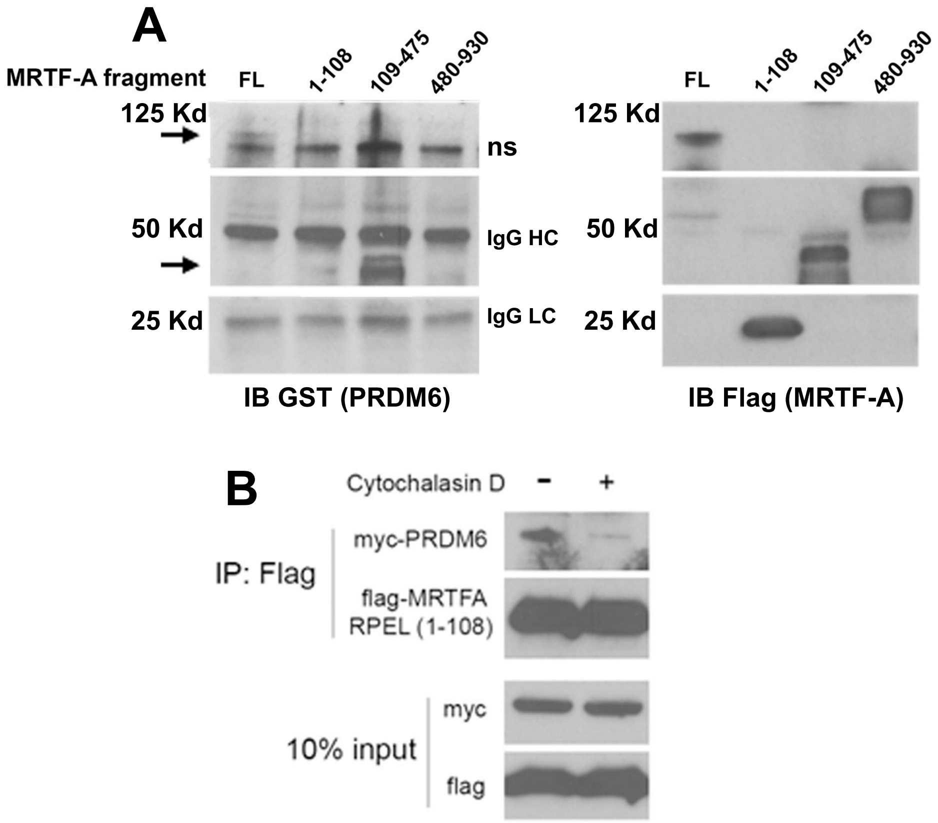

Supplemental Figure 1. Prdm6 interacted directly with MRTF-A. A) The indicated Flag-MRTF-A fragments were expressed in Cos-7 cells, purified by immunoprecipitation, run on an SDS page gel, and transferred to nitrocellulose. Following renaturation, blots were incubated with GST-Prdm6, washed, and then probed with an anti-GST Ab. Arrows mark interactions between PRDM6 and full length and 109-475 MRTF-A variants. n=2, representative blot shown. B) A flag-tagged N-terminal fragment of MRTFA (AA1-108) and full length myc-Prdm6 were expressed in COS cells. The MRTF-A N-terminal fragment was immunoprecipitated in the presence and absence of the actin binding drug cytochalasin, and washed immunoprecipitates were probed for myc. n=2, representative blot shown.

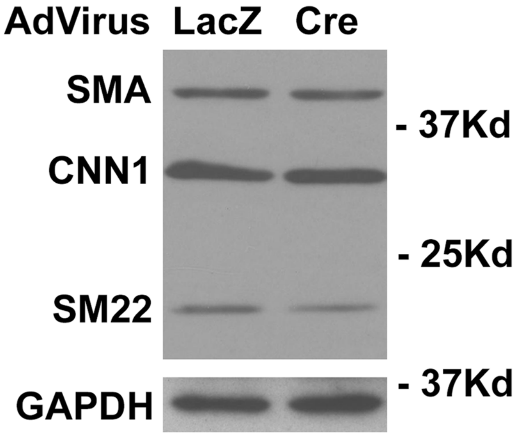

Supplemental Figure 2. PRDM6 depletion inhibited SMC marker protein expression. Outflow tract SMCs isolated from Prdm6 flox/flox mice were treated with Cre or LacZ expressing virus. RIPA lysates were run on a 10% SDS page gel, transferred to nitrocellulose, and then probed for the indicated SMC marker. n=2, representative blot shown.

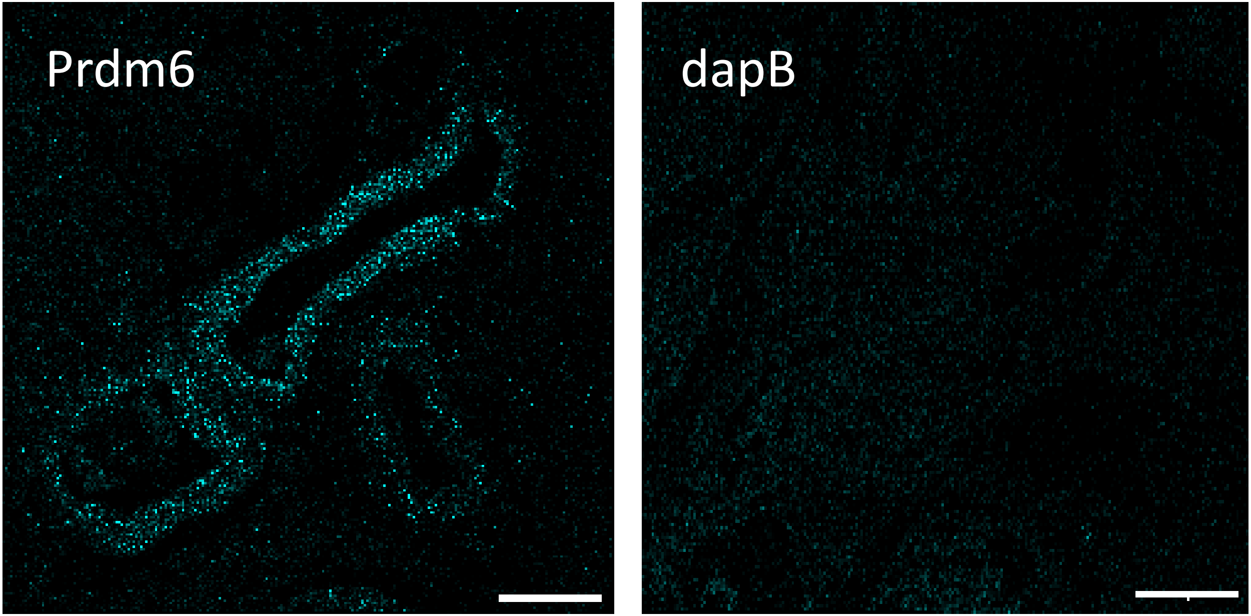

Supplemental Figure 3. Formalin fixed, paraffin embedded sections through the ductus arteriousus from E18.5 mice were processed for RNAscope-based in situ hybridization using probes specific to Prdm6 (left) or the bacterial gene, dapB (right). Scale bar = 200 microns

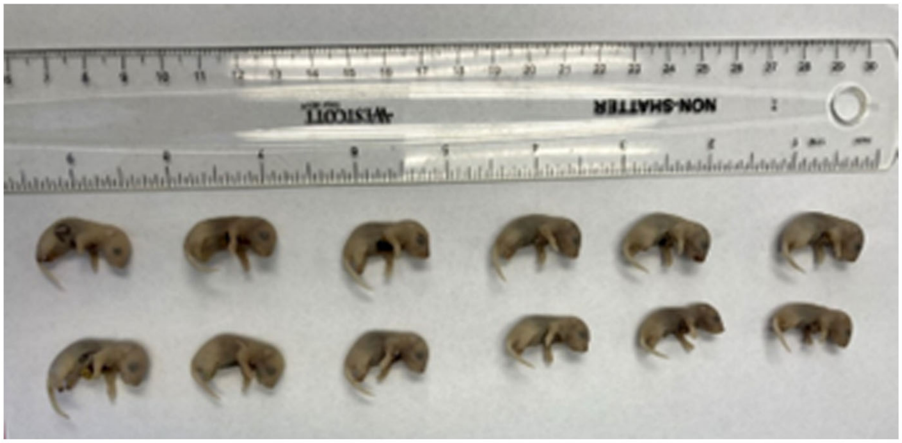

Supplemental Figure 4. Neural crest cell-specific deletion of Prdm6 did not affect the overall size or general appearance of P1 mouse pups. Wnt1Cre2PRDM6 flox/flox P1 pups (top) and littermate control P1 pups (bottom) from crosses between male and female Wnt1Cre2PRDM6 Wt/flox mice. Note that the littermate control group consists of the following genotypes - Wnt1Cre2PRDM6 Wt/flox, Wnt1Cre2PRDM6 Wt/Wt, PRDM6 flox/flox , PRDM6 Wt/flox, and PRDM6 Wt/Wt.

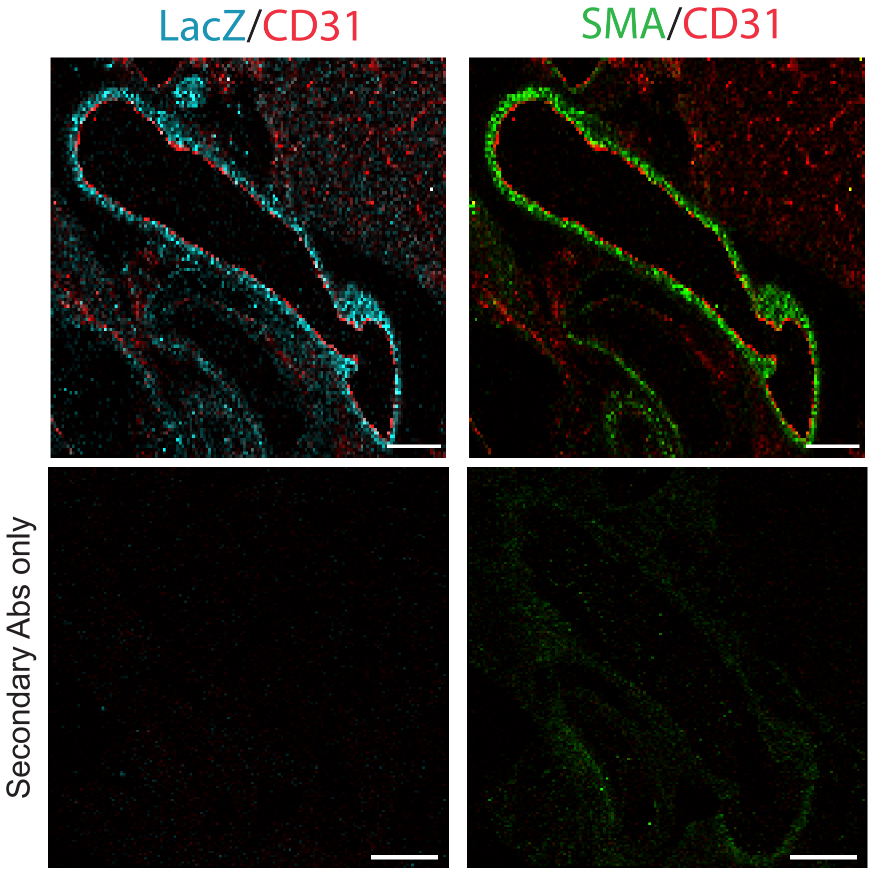

Supplemental Figure 5. Strong overlap between LacZ and SM α-actin expression in outflow tract SMC of Wnt1Cre2ROSA26LacZ mice. Formalin fixed, paraffin embedded sections through the aortic arch of Wnt1Cre2ROSA26LacZ P1 mice were stained with anti-LacZ, anti-SM α-actin (SMA), and anti-CD31 Abs. Note the strong overlap between LacZ and SM α-actin expression and that we observed little to no background staining with secondary Abs alone.

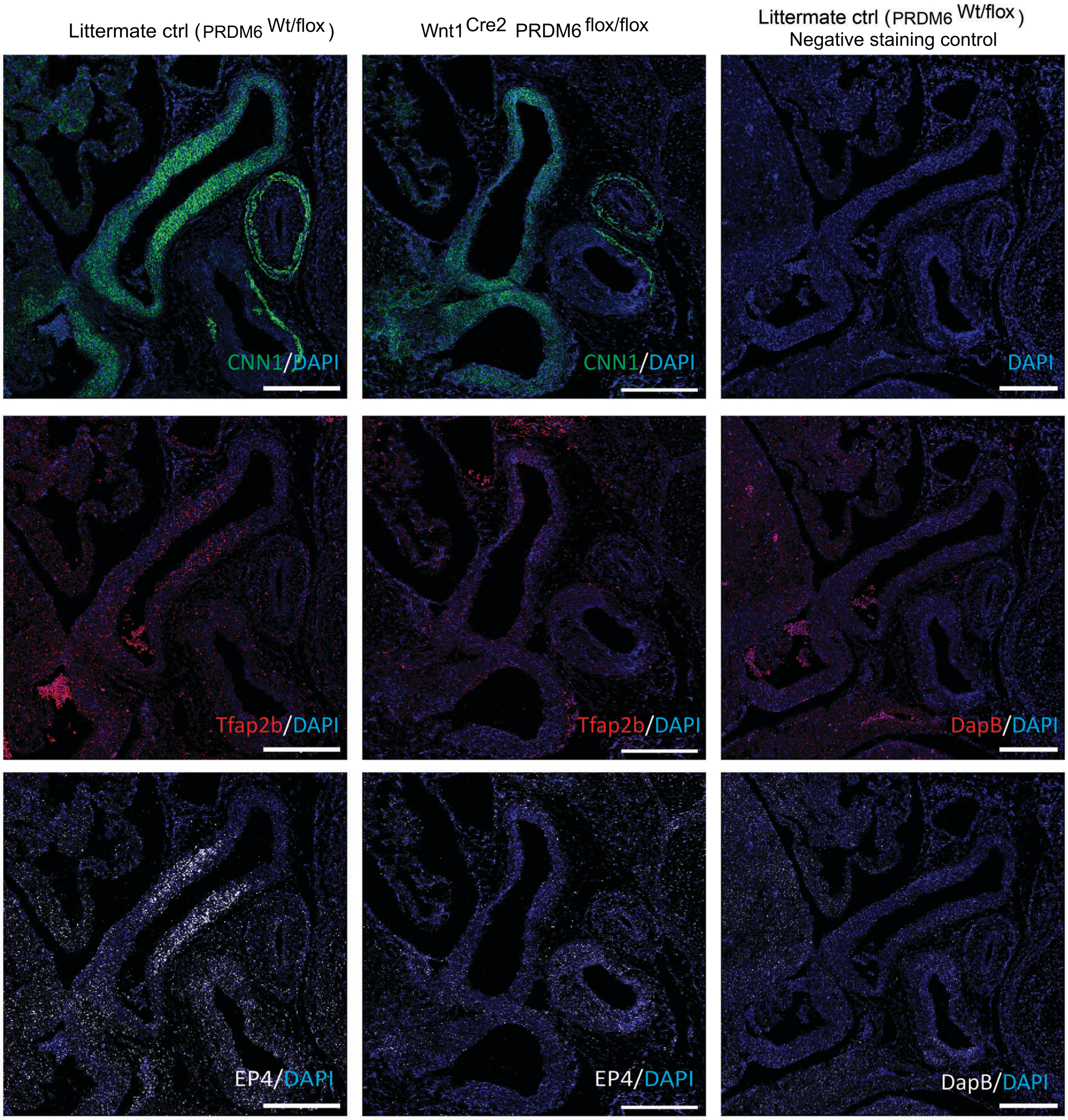

Supplemental Figure 6. PRDM6 depletion in neural crest-derived SMC inhibited the expression of EP4, Tfap2b, and CNN1. Formalin fixed, paraffin embedded sections through the ductus arteriousus of Wnt1Cre2Prdm6flox/-flox and littermate control mice at E18.5 were processed for immunofluorescence-based detection of CNN1 (top), RNAscope-based detection of Tfap2B (middle), or RNAscope-based detection of EP4 expression (bottom). Note the lack of staining with secondary Ab only or with the use of an RNAscope in situ probe against the bacterial protein dapB (right column).

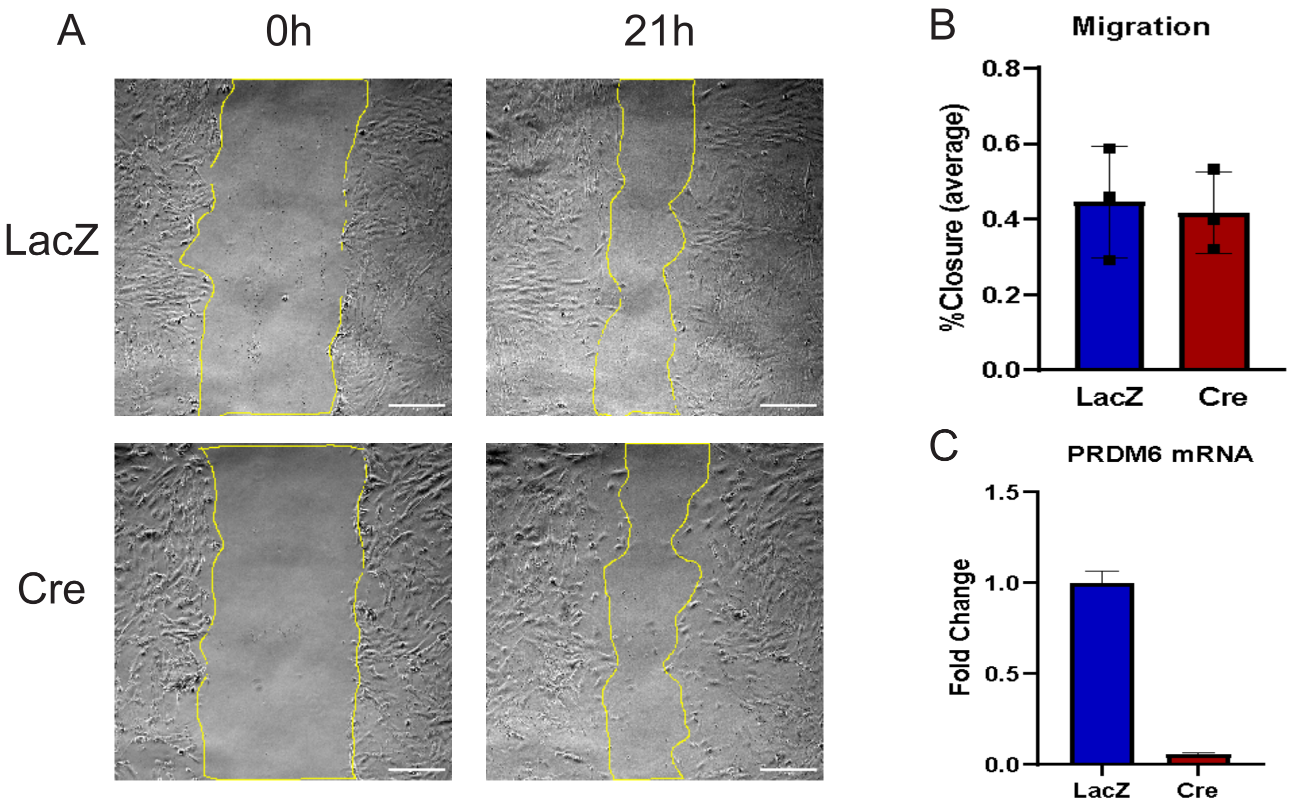

Supplemental Figure 7. PRDM6 depletion did not affect migration of cultured outflow tract SMCs. A) Outflow tract SMCs isolated from Prdm6 flox/flox mice were treated with Cre or LacZ expressing virus, grown to confluence, and then subjected to scratch wounding as shown. B) Wound closure at 21 h was quantified by averaging wound diameter measurements taken from 10 different locations. n=3. C) Prdm6 expression in Cre-and LacZ-infected cells was measured by qPCR.
